# The neuromodulator of exploration: A unifying theory of the role of dopamine in personality

**DOI:** 10.3389/fnhum.2013.00762

**Published:** 2013-11-14

**Authors:** Colin G. DeYoung

**Affiliations:** Department of Psychology, University of MinnesotaMinneapolis, MN, USA

**Keywords:** dopamine, personality, extraversion, openness, impulsivity, sensation seeking, depression, schizotypy

## Abstract

The neuromodulator dopamine is centrally involved in reward, approach behavior, exploration, and various aspects of cognition. Variations in dopaminergic function appear to be associated with variations in personality, but exactly which traits are influenced by dopamine remains an open question. This paper proposes a theory of the role of dopamine in personality that organizes and explains the diversity of findings, utilizing the division of the dopaminergic system into value coding and salience coding neurons (Bromberg-Martin et al., 2010). The value coding system is proposed to be related primarily to Extraversion and the salience coding system to Openness/Intellect. Global levels of dopamine influence the higher order personality factor, Plasticity, which comprises the shared variance of Extraversion and Openness/Intellect. All other traits related to dopamine are linked to Plasticity or its subtraits. The general function of dopamine is to promote exploration, by facilitating engagement with cues of specific reward (value) and cues of the reward value of information (salience). This theory constitutes an extension of the entropy model of uncertainty (EMU; Hirsh et al., 2012), enabling EMU to account for the fact that uncertainty is an innate incentive reward as well as an innate threat. The theory accounts for the association of dopamine with traits ranging from sensation and novelty seeking, to impulsivity and aggression, to achievement striving, creativity, and cognitive abilities, to the overinclusive thinking characteristic of schizotypy.

Personality neuroscience is an interdisciplinary approach to understanding mechanisms in the brain that produce relatively stable patterns of behavior, motivation, emotion, and cognition that differ among individuals (DeYoung and Gray, 2009; DeYoung, 2010b). Dopamine, a broadly acting neurotransmitter, is one of the most studied and theorized biological entities in personality neuroscience. Dopamine acts as a neuromodulator; relatively small groups of dopaminergic neurons in the midbrain extend axons through much of the frontal cortex, medial temporal lobe, and basal ganglia, where dopamine release influences the function of local neuronal populations. Despite the extensive attention paid to dopamine in personality neuroscience, no comprehensive theory exists regarding its role in personality, and it has been implicated in traits ranging from extraversion to aggression to intelligence to schizotypy.

The present article attempts to develop a unifying theory to explain dopamine's apparently diverse influences on personality, linking it to all traits that reflect variation in processes of exploration. Exploration is defined as any behavior or cognition motivated by the incentive reward value of uncertainty. (This definition will be explored in more detail below, in the section titled *Exploration, Entropy, and Cybernetics*.) Personality traits can be explained as relatively stable responses to broad classes of stimuli (Tellegen, 1981; Gray, 1982; Corr et al., 2013). Personality traits associated with dopamine, therefore, are posited to be those that reflect individual differences in incentive responses to uncertainty.

## Dopamine as driver of exploration

Before discussing personality traits in detail, it will be necessary to have a working model of dopaminergic function. In my attempt to develop a unifying theory of the role of dopamine in personality, I also posit a unifying theory of the function of dopamine in human information processing. One might think it naïve to assume that complex neuromodulatory systems have any core function unifying their diverse processes. Dopamine is involved in a variety of cognitive and motivational processes; dopaminergic neurons originate in multiple sites in the midbrain; and dopaminergic axons extend to multiple regions of the striatum, hippocampus, amygdala, thalamus, and cortex. Finally, there are five different dopamine receptors, in two classes (D1 and D5 are D1-type, whereas D2, D3, and D4 are D2-type), with very different distributions in the brain. Why should not this diversity have evolved to serve several independent functions, with no unifying higher-order function? The simple reason this seems unlikely is evolutionary path-dependency. If dopamine served a particular function in a phylogenetically early organism, then it would be easier for evolution to co-opt the dopaminergic system to perform additional functions if they were not incompatible with the first function, and easier still if the new functions were influenced by some broad selective pressure that also influenced the older function, which is to say, if they shared some more general function. This is because any factor that affects synthesis of dopamine, whether genetic, metabolic, or dietary/digestive, is likely to influence all aspects of dopaminergic function, no matter how diverse, as it will tend to increase or decrease available dopamine in all branches of the system. The maintenance of some overarching consistency of dopaminergic function by evolution is likely because it would avoid conflict between different branches of the system when global levels of dopamine are raised or lowered. Note that this is an argument about what is evolutionarily *likely*, not what is evolutionarily necessary; it is intended merely as preliminary evidence for the plausibility of the unifying theory that follows.

The nature of evolutionary path-dependency suggests a hierarchical organization of functions of the dopaminergic system. The different functions carried out by different branches and components of the dopaminergic system are posited, in the present theory, to have one higher-order function in common, and that function is exploration. The release of dopamine, anywhere in the dopaminergic system, increases motivation to explore and facilitates cognitive and behavioral processes useful in exploration.^1^

Different forms of exploration exist, however, and these are governed by different subsystems of the dopaminergic system. Further, different branches of the dopaminergic system are likely to have different effects on different brain regions (e.g., cortical vs. subcortical regions) in order to adjust neural populations in those regions to particular functional demands. Thus, the dopaminergic system can be considered to carry out multiple distinct functions, which may appear extremely diverse or even incompatible when considered at the level of specific brain structures, but which nonetheless possess a larger functional unity.

### Exploration, entropy, and cybernetics

Before providing evidence that this functional unity reflects exploration, the definition of exploration as “any behavior or cognition motivated by the incentive reward value of uncertainty” must be explained. To explore is to transform the unknown into the known or the known into the unknown (Peterson, 1999). More formally, what is unknown is what is uncertain or unpredicted, and what is uncertain or unpredicted can be defined in terms of psychological entropy^2^. The theory I present here is an extension of the entropy model of uncertainty (EMU), which posits that anxiety is a response to psychological entropy (Hirsh et al., 2012). Entropy is a measure of disorder, originally developed to describe physical systems (Clausius, 1865; Boltzmann, 1877) but later generalized to all information systems (Shannon, 1948). It can be most simply defined as the number of microstates possible in a given macrostate. For example, the entropy of a shuffled deck of cards is a function of the number of possible sequences of cards in the deck; in contrast, the entropy of a new, unopened deck of cards is much lower, because decks of cards ship with their suits together in numerical order. Entropy, therefore, describes the amount of uncertainty or unpredictability in an information system. Human beings are complex information systems, and, specifically, they are cybernetic systems—that is, goal-directed, self-regulating systems (Carver and Scheier, 1998; Peterson and Flanders, 2002; Gray, 2004; Van Egeren, 2009; DeYoung, 2010c). Wiener (1961), the founder of cybernetics, noted that the entropy of a cybernetic system reflects the uncertainty of its capacity to move toward its goals at any given time.

As a cybernetic system, the human brain must encode information about (1) desired end states or goals, (2) the current state, largely comprising evaluations and representations of the world as it is relevant to those goals, and (3) a set of *operators* potentially capable of transforming the current state into the goal state; operators are skills, strategies, and plans that aid one in moving toward one's goals (Newell and Simon, 1972; DeYoung, 2010c). (All of these may be encoded both consciously and unconsciously. In psychology, the term “goal” is sometimes reserved for explicit, conscious, specific formulations of goals, but the term is used here in the broader, cybernetic sense.) The amount of uncertainty in these three cybernetic elements of a person constitutes *psychological entropy*, which reflects the number of plausible options or affordances available to the individual for representation (both perceptual and abstract) and for behavior, at any given time (Hirsh et al., 2012). In other words, the harder it is for the brain to answer the questions, “What is happening?” and “What should I do?” the higher the level of psychological entropy. Again, the brain addresses these questions both consciously and unconsciously; thus, they need not be explicitly framed in language to be a constant feature of human psychological functioning.

In explicating EMU, Hirsh et al. (2012) described anxiety as the innate response to increases in psychological entropy. Entropy is necessarily aversive to a cybernetic system because it renders the function of that system (progress toward its goals) more difficult. In other words, uncertainty is threatening. The crucial extension of EMU developed in the present theory is that, although entropy is innately aversive, it is simultaneously innately incentively rewarding. In fact, what is uncertain or unpredicted is unique as a class of stimuli in being simultaneously threatening and promising (Peterson, 1999; Peterson and Flanders, 2002). This unusual, ambivalent property of unpredicted or novel stimuli has been well-established in research on reinforcement learning (Dollard and Miller, 1950; Gray and McNaughton, 2000), and can be grasped intuitively by considering instances in which people seek out uncertainty for the excitement it provides, despite attendant risk or even the expectation that loss is more likely than gain (e.g., gambling).

In cybernetic terms, rewards are any stimuli that indicate progress toward or attainment of a goal, whereas punishments are any stimuli that disrupt progress toward a goal. These definitions are generally compatible with the behaviorist definition of rewards and punishments as stimuli that increase or decrease, respectively, the frequency of the behaviors leading up to them. Two classes of reward can be distinguished: consummatory rewards, which represent the actual attainment of a goal, and incentive rewards, also called cues of reward or promises, which indicate an increase in the probability of achieving a goal. Similarly, one can distinguish between punishments, which represent definite inability to reach a goal, and threats, or cues of punishment, which indicate a decrease in the likelihood of achieving a goal. (Note that goals can be of any level of abstraction, ranging from concrete goals like avoiding pain to abstract goals like succeeding in business, falling in love, or understanding Joyce's *Ulysses*.) Importantly, because of the nested nature of goals, in which superordinate goals are achieved through the accomplishment of more immediate subgoals, a single stimulus can be simultaneously a punishment and a threat (of further punishment) or simultaneously a consummatory reward (attainment of a subgoal) and an incentive reward (cuing increased likelihood of attaining the superordinate goal).

The reason that increases in psychological entropy are threatening is relatively obvious, whereas the reason that they are simultaneously promising is probably not. How could an increase in entropy simultaneously indicate decreased and increased likelihood of meeting one's goals? The most basic and general answer is that an unpredicted event signals uncertainty about the likelihood of meeting one's goals. This likelihood may be increased or decreased depending on the as-yet-undetermined implications of the unpredicted event. (Remember, as well, that people have multiple goals, and an unpredicted event may increase the likelihood of reaching one goal even as it decreases the likelihood of reaching another.) Another way to say this is that everything both good and bad comes initially out of the unknown, so that an unpredicted event may signal an obstacle or an opportunity (or it may simply be neutral, signaling nothing of relevance to any goal), and which of these possibilities is signaled is often not immediately evident (Peterson, 1999). What this implies is that the organism should have two competing innate responses to an unpredicted event—caution and exploration—and this is exactly what has been demonstrated (Gray and McNaughton, 2000). (Here it is important to note that “unpredicted” can refer to any aspect of an event, such that an event of interest can be unpredicted, even if it is strongly expected, as long as its timing is not perfectly predicted). Animals have evolved a suite of behaviors useful in situations in which they do not know exactly what to do or what to think—in other words, when prediction fails. Some of these behaviors are defensive, as what you don't know *can* hurt you, and some are exploratory, as an uncertain situation might always include some as yet undiscovered reward.

### Types of uncertainty and the reward value of information

Unpredicted events are unified functionally by the fact that they increase psychological entropy. Nonetheless, they vary widely in the degree and manner in which they do so, and this variation helps to determine whether caution or exploration will predominate in response to any given anomaly. For many unpredicted stimuli, it will be quickly evident that they signal a specific reward or punishment (or something definitely neutral, which requires no response beyond learning the irrelevance of the stimulus). In the case of reward, psychological entropy may be increased relatively little, and the optimal response is often straightforward: First, in all cases of unpredicted reward, learning should take place, both so that the behavior that led to the reward is reinforced and so that environmental cues that may predict the reward are remembered. This learning constitutes a very basic form of cognitive exploration, transforming the unknown into the known and the unpredictable into the predictable. Second, if the unpredicted stimulus is an incentive reward rather than a consummatory reward, additional approach behavior will often be necessary to attempt to attain the consummatory reward that is signaled. The effort expended in this attempt is exploratory (and accompanied by heightened dopamine release) to the degree that attainment of the reward remains uncertain following the cue (Schultz, 2007). The one condition—a fairly common occurrence—that makes the increased entropy accompanying unexpected incentive reward more than minimal is when pursuing the reward would disrupt the pursuit of some other currently operative goal. As discussed in the next section, one division of the dopaminergic system appears to potentiate both reinforcement learning and approach behavior in response to unpredicted reward.

In the case of unpredicted stimuli that signal a specific punishment, determination of what to do is more complicated, primarily because punishments or negative goals are repulsors rather than attractors (Carver and Scheier, 1998). Attractors are goals that require a cybernetic system to minimize distance between current state and desired state. Repulsors, in contrast, require increasing the distance of the current state from the undesired state, but they do not inherently specify a concurrent attractor that could guide behavior. Thus, psychological entropy is typically increased more by unexpected punishment than by unexpected reward. As a general rule, the greater the increase in entropy, the more likely aversion is to predominate over exploration (Peterson, 1999; Gray and McNaughton, 2000). Nonetheless, the present theory argues that all uncertainty has incentive value, and unpredicted threat or punishment is the crucial test case. What is the incentive reward value of an unexpected event that clearly signals a specific punishment? Put simply, one potential consummatory reward signaled by any unpredicted event is information, which is identical to a decrease of psychological entropy. Exploration is worthwhile, even in the case of an unexpected punishment, because it may lead to an increase of information, which will allow the person to better represent the world or select behavior in future, which in turn increases the likelihood of goal attainment (and the relevant goal may simply be avoiding the punishment in question). In other words, any unpredicted event, including unpredicted threat or punishment, signals the possibility that exploration may lead to a rewarding decrease in psychological entropy. In the case of threat, cognitive exploration (searching for relevant patterns in perception and memory) is more likely to be adaptive than approach-oriented behavioral exploration because a known punishment should usually be avoided rather than approached. As discussed below, the other major division of the dopaminergic system appears to potentiate exploration in response to the incentive value of the possibility of gaining information—that is, it drives curiosity or desire for information.

Information potentially relevant for optimal adjustment of the parameters of a cybernetic system logically has reward value for that system. Empirical evidence is consistent with this assertion. Bromberg-Martin et al. (2010) cite several studies that have shown both humans and other species to have a preference for environments in which rewards, punishments, and even neutral sensory events can be predicted in advance—in other words, environments with greater available information (Badia et al., 1979; Daly, 1992; Chew and Ho, 1994; Herry et al., 2007). Further, they have shown that dopaminergic activity tracks this preference in monkeys (Bromberg-Martin and Hikosaka, 2009). This preference is adaptive for any cybernetic system that can utilize information about its environment to predict an effective course of action in any given situation. The fact that a preference exists even for neutral events to be predictable is of interest because it illustrates the fact that information is rewarding even if it is not immediately connected to a known reward or punishment. This is sensible because, in any naturalistically complex environment, what is neutral or irrelevant at present may become motivationally relevant in future. Thus, the information about the present state maintained by the cybernetic system is likely to include some potentially extraneous detail, not inherently linked to a currently operative goal. Another demonstration of the reward value of information comes from two studies of curiosity, utilizing trivia questions (Kang et al., 2009). A functional magnetic resonance imaging (fMRI) study showed that neural reward signals in the dorsal striatum, upon seeing the answer to trivia questions, were correlated with the amount of curiosity about the answer. Thus, desired information triggers the brain's reward system in much the same way that monetary, social, or food rewards do. A second study showed that people are willing to expend limited resources to acquire answers to trivia questions, much as they are to acquire more concrete rewards.

The third important category of unpredicted stimuli is also clearly linked to the reward value of information; these are stimuli in which what is signaled is itself uncertain. Whether they are threatening, promising, or neutral is ambiguous, at least initially. When such stimuli are proximal or otherwise particularly salient (e.g., a loud, unexpected noise nearby), they trigger an alerting or orienting response, which involves the involuntary direction of attention toward the stimulus, so as to aid in identifying its significance (Bromberg-Martin et al., 2010). This is a reflexive form of exploration, aimed at acquiring information (and potentially capturing fleeting reward). Obviously, unpredicted stimuli of ambiguous value are not a discrete category but exist on a continuum with the unpredicted stimuli (described above) that quickly and clearly signal specific rewards or punishments. The more ambiguous the unpredicted stimulus, the more strongly it should drive both cognitive and behavioral exploration. However, the larger its magnitude as an anomaly—that is, the more psychological entropy it generates, which is a function of which goals and representations it disrupts—the more strongly it will also drive defensive aversion responses, including caution, anxiety, fear, or even panic (Peterson, 1999; Gray and McNaughton, 2000). Severely anomalous events, which have highly uncertain meaning, constitute one of the most motivating but also the most conflict-generating, and thus stressful, classes of stimuli. They trigger massive release of neuromodulators, including both dopamine, to drive exploration, and noradrenaline (also called “norepinephrine”), to drive aversion and to constrain exploration (Robbins and Arnsten, 2009; Hirsh et al., 2012).

Although dopamine is the focus of the present theory, it will be necessary to refer occasionally to noradrenaline, which is posited by EMU as the major neuromodulator of anxiety (Hirsh et al., 2012). Noradrenaline has been described as a response to “unexpected uncertainty” that acts as an “interrupt” or “stop” signal following increases in psychological entropy (Aston-Jones and Cohen, 2005; Yu and Dayan, 2005). The release of noradrenaline in response to uncertainty leads to increased arousal and vigilance and to slowing or interruption of ongoing goal directed activity. Noradrenaline is released in both phasic and tonic firing patterns. Short phasic bursts of noradrenaline are necessary for appropriate flexibility within a task, allowing switching between different strategies and representations when the need arises (Robbins and Roberts, 2007). Tonic elevations in noradrenaline, however, appear to indicate a more persistent increase in psychological entropy and increase the likelihood that performance in a task will be slowed or interrupted, often with concurrent anxiety (Aston-Jones and Cohen, 2005; Hirsh et al., 2012). Whereas dopamine is posited to signal the incentive value of uncertainty, noradrenaline signals the aversive value of uncertainty (which, in a cybernetic framework, is equivalent to the degree that uncertainty should disrupt ongoing goal-directed action). Thus, the present theory holds that dopamine and noradrenaline act in competition in response to uncertainty, setting the balance between exploration and aversion.

### Functional neuroanatomy of the dopaminergic system

The dopaminergic system appears to be largely organized around two classes of incentive motivation: the incentive reward value of the possibility of specific goal attainment, and the incentive reward value of the possibility of gains in information. The theory developed here is based heavily on a model of the dopaminergic system proposed by Bromberg-Martin et al. (2010), who reviewed and synthesized a great deal of what is known about dopamine into a coherent model positing two distinct types of dopaminergic neuron, which respond to three different types of input. The two types of dopaminergic neuron they label *value coding* and *salience coding*. Value coding neurons are activated by unpredicted reward and inhibited by unpredicted aversive stimuli (including omission of expected reward). The magnitude of their activation reflects the degree to which the value of the stimulus over- or under-shoots expectations. They thus provide a signal of the value of unpredicted stimuli. Salience coding neurons are activated by unpredicted punishments as well as unpredicted rewards and thus provide an index of the salience, or degree of motivational significance, of stimuli. In addition to value and salience signals, a third type of input, consisting of *alerting signals*, excites both value coding and salience coding neurons (there do not appear to be any distinct “alerting neurons”). Alerting signals are responses to any “unexpected sensory cue that captures attention based on a rapid assessment of its potential importance” (Bromberg-Martin et al., 2010, p 821) and correspond to the third category of unpredicted stimuli discussed above, in which the value of a stimulus is initially unclear.

Where the present theory extends the theory of Bromberg-Martin et al. (2010) is in positing that both value coding and salience coding dopaminergic neurons are driven by unpredicted incentives specifically, and that all dopamine release potentiates exploration designed to attain the rewards signaled by those incentives. The hypothesis that the dopaminergic system responds to unpredicted incentive rewards is not new (e.g., Schultz et al., 1997; Depue and Collins, 1999); however, previous theories of incentive reward applied only to value coding dopaminergic neurons. According to the present theory, salience coding neurons respond to incentive cues for the value of information that can potentially be obtained following any increase in psychological entropy, regardless of whether this increase stems from an unexpected reward, an unexpected punishment, or a stimulus of unknown value. The recognition that information itself has incentive value for a cybernetic system allows the integration of both divisions of the dopaminergic system into a unified theoretical framework, in which the overarching function of the whole dopaminergic system can be identified as the potentiation of exploration. Despite this abstract functional commonality, however, the differences between the value and salience coding divisions of the dopaminergic system are extensive and crucial for understanding dopaminergic function and its role in personality. Thus, I next summarize the functional neuroanatomy of the two divisions of the dopaminergic system, as described primarily by Bromberg-Martin et al. (2010).

Dopaminergic neurons are primarily concentrated in two adjacent regions of the midbrain, the ventral tegmental area (VTA) and the substantia nigra pars compacta (SNc). (In the primate brain, dopaminergic neurons have recently been discovered that project to the thalamus from several regions other than VTA and SNc, but much less is known about these; Sánchez-González et al., 2005.) The distribution of value coding and salience coding neurons forms a gradient between VTA and SNc, with more value coding neurons in the VTA and more salience coding neurons in the SNc. Nonetheless, populations of both types of neurons are present in both areas. From the VTA and SNc, dopaminergic neurons send axons to release dopamine in many brain regions, including the basal ganglia, frontal cortex, extended amygdala, hippocampus, and hypothalamus. Bromberg-Martin et al. (2010) present evidence that value coding neurons project preferentially to the shell of the nucleus accumbens (NAcc) and the ventromedial prefrontal cortex (VMPFC), whereas salience coding neurons project preferentially to the core of the NAcc and the dorsolateral PFC (DLPFC). Both value and salience coding neurons project to the dorsal striatum (caudate and putamen). For other brain structures, it is currently unclear whether they are innervated by value or salience coding neurons. Dopamine release in the amygdala increases during stress (the presence of aversive stimuli), which is likely to indicate activity of the salience system specifically (Pezze and Feldon, 2004). The anatomical distribution of projections from value vs. salience neurons renders each type of neuron appropriate to produce different types of response to uncertainty, which can be described as different forms of exploration. This is particularly evident in relation to the neuroanatomical structures currently known to be uniquely innervated by each type of dopaminergic neuron.

Value coding neurons are described by Bromberg-Martin et al. (2010) as supporting brain systems for approaching goals, evaluating outcomes, and learning the value of actions. These processes are involved in exploration for specific rewards. The VMPFC is crucial for keeping track of the value of complex stimuli, and the shell of the NAcc is crucial to engagement of approach behavior and reinforcement of rewarded action. Additionally, in the dorsal striatum, a detailed model exists describing how the value system signals values both better and worse than predicted. Dopaminergic neurons have two primary modes of firing: a tonic mode, in which, as their default, they fire at a relatively constant, low rate, and a phasic mode, in which they fire in bursts at a much higher rate in response to specific stimuli. Value coding dopaminergic neurons have also been demonstrated to show phasic reductions in firing, below the tonic baseline, in response to outcomes that are worse than predicted (as in omission of expected reward), which enables them to code negative as well as positive values. Whereas phasic responses in the value system signal the value of unpredicted stimuli, shifts in tonic level have been hypothesized to track the long-run possibilities for reward in a given situation and to govern the vigor or energy with which an individual acts (Niv et al., 2007); in the present theory, the tonic level would correspond to the general strength of the exploratory tendency, in contrast to the exploratory responses to specific stimuli produced by phasic bursts of dopamine. Phasic increases and decreases in firing by the value system interact with two different dopamine receptor subtypes in the dorsal striatum to transform the value signal into either facilitation or suppression of exploratory approach behavior, depending on the presence of unpredicted rewards or punishments (Bromberg-Martin et al., 2010; Frank and Fossella, 2011).

Salience coding neurons are described by Bromberg-Martin et al. (2010) as supporting brain systems for orienting of attention toward motivationally significant stimuli, cognitive processing, and increasing general motivation for any relevant behavior, processes that are involved in exploration for information. The DLPFC is crucial for working memory, which involves the maintenance and manipulation of information in conscious attention and is thus central to most complex cognitive operations. Adequate dopamine in DLPFC is crucial for maintaining representations in working memory (Robbins and Arnsten, 2009). The core of the NAcc is important for overcoming the cost of effort, for enhancement of general motivation, and for some forms of cognitive flexibility (Bromberg-Martin et al., 2010). The theory presented here hinges on the premise that, whereas the value system is designed to potentiate behavioral exploration for specific rewards, the salience system is designed to potentiate cognitive exploration for information.

In considering individual differences in personality related to the dopaminergic system, I argue that the most important distinction is between value and salience coding dopaminergic neurons. Of course, the dopaminergic system contains many further complexities that are likely to have important consequences for individual differences in behavior, motivation, emotion, and cognition. These include the difference between tonic and phasic firing patterns, different receptor types, and differences in mechanisms of reuptake and synaptic clearance in different brain regions, among many others. Regarding how these differences influence specific traits, however, too little evidence exists to be of much use. At the level of resolution with which personality neuroscience has been studied to date, the difference between the value and salience coding systems appears to be sufficient to create a relatively unified account of how dopamine is involved in personality. Hopefully, future research will flesh out the framework presented here with a more detailed model of how more fine-grained differences within each of the two major divisions of the dopaminergic system influence personality.

### Exploration: motivation and emotion associated with dopamine

With a basic understanding of dopaminergic neuroanatomy, we can now turn to the question of how dopaminergic function is manifest in human behavior and experience. To say that it is manifest in exploration is likely to be misleading without a thorough understanding of the pervasive influence of the exploratory tendency. Some might argue that my use of “exploration” to describe all cognition and behavior in response to the incentive reward value of uncertainty is problematically broad, but this breadth is crucial to the theory. The assertion that all dopaminergic function is in service of exploration hinges on the observation that dopamine is not released in response to all motivationally relevant stimuli (e.g., all cues of reward), but only to those that are unpredicted or uncertain. Thus, dopamine is not simply an energizer of all behavior. Indeed, Ikemoto and Panksepp (1999, p 24) argued that “the effects of [dopamine] agonists may be better characterized as elevations in general exploration rather than general motor activity.”

Following Peterson (1999), I argue that all psychological function is either engaged with the unknown (adapting to increases in psychological entropy through exploration), or it is concerned with stabilizing ongoing goal pursuit (engaging in activities aimed at preventing increases in psychological entropy)^3^. This observation highlights the continual necessity of exploration, as uncertainty arises frequently across a wide range of magnitudes of implication for representation and behavior. For minor uncertainties, processes of exploration are unlikely to be conscious or explicitly noted using the colloquial vocabulary of “exploration,” but they are nonetheless importantly exploratory in their function. For example, many processes of learning can be considered exploration. (To equate all processes of learning with exploratory processes potentiated by dopamine would be too broad, however. Learning from punishment, for example, often involves contraction of the cybernetic system, abandoning a particular goal or subgoal and avoiding it in future. This kind of learning as pruning of the goal system is specifically punishment-related and probably facilitated by noradrenaline rather than dopamine.) Any kind of expansive rather than contractive learning, in which new associations are being formed, is exploratory and probably facilitated by dopamine (Knecht et al., 2004; Robbins and Roberts, 2007).

Another case in which some might consider my use of the term “exploration” too broad comes in contexts where exploration has been contrasted with exploitation (Cohen et al., 2007; Frank et al., 2009). These are situations in which the individual must choose between continuing to pursue a strategy with a reward value that is at least partly predictable (exploitation), or switching to some other strategy with an unknown reward value that may be greater (but may be less) than that of the current strategy (exploration). This is an important distinction, but I would argue that, even in exploitation mode, some forms of dopaminergically mediated exploration take place, unless the reward in question and its associated cues are entirely predictable, in which case no dopaminergic activity will be evoked. This exploration includes not only learning about the reward and its cues but also any effort exerted to ensure the delivery of the reward, as long as that delivery is at all uncertain. One crucial fact about the dopaminergic system is that its tonic activity increases following a cue of reward, in proportion to the degree that delivery of that reward remains uncertain, and this increase is distinct from the phasic bursts that accompany unpredicted reward or cues of reward (Schultz, 2007). This tonic elevation seems likely to occur to potentiate effort that could increase the likelihood of acquiring uncertain rewards, and, given the premise the dopamine always potentiates exploration, it supports the existence of exploratory processes during most cases of “exploitation.” Finally, although the switch from exploitation mode to exploration mode may be accomplished by noradrenergic interruption of goal directed activity (Cohen et al., 2007), once the individual is in exploration mode, dopaminergic activity in both value and salience systems should increase to facilitate exploratory behavior (Frank et al., 2009).

What are the motivational states that accompany exploration? Activity in the value coding system should be accompanied by motivation (conscious or unconscious) to learn how stimuli and actions predict reward and to exert vigorous effort to reach goals. Activity in the salience coding system should be accompanied by motivation to learn what predicts reward or punishment and to engage cognitive effort to understand the correlational and causal structure of relevant stimuli. When both systems are activated together by an alerting stimulus, they should produce strong motivation to learn what just happened and to exert cognitive and motor effort to classify the unpredicted event.

Note that in the case of unexpected reward, both value and salience coding dopaminergic neurons will typically be activated. This is sensible because of the potential benefit from exploring both the possibility of acquiring the specific reward in question (signaled by value neurons) and the possibility of gaining information about the reward and its context (signaled by salience neurons). In the case of unexpected punishment, however, salience neurons will be activated, whereas value neurons will be suppressed. This should facilitate general motivation to cope with the threat and cognitive and perceptual exploration of the situation, while suppressing behavioral exploration that might be risky. The general motivation produced by the salience system may, in the presence of aversive stimuli, aid in overcoming the cost of effort to explore possible coping strategies for dealing with the threat. Overcoming the cost of effort appears to be an important function of dopamine, probably attributable to the value system as well as the salience system. This was demonstrated by a recent study showing that individual differences in dopaminergic function in the striatum and VMPFC predicted willingness to expend effort to seek reward, particularly when probability of receiving the reward was low (Treadway et al., 2012).

Dopamine produces motivation to exert effort to seek reward or information, but this does not entirely clarify what emotions accompany dopamine release. Because of its role in response to reward, dopamine has often been erroneously described as a “feel-good” chemical. There is no doubt that dopamine can make people feel good; drugs that increase dopaminergic function, like cocaine or amphetamine, are abused in part because they produce feelings of excitement, elation, and euphoria. In neuroimaging studies, degree of self-reported elation in response to cocaine was associated with dopaminergic response and levels of neural activity in the striatum (Breiter et al., 1997; Volkow et al., 1997). Increasingly, however, research shows that positive hedonic tone, the pleasure or liking felt for reward, is not directly due to dopamine, but rather to other neurotransmitters, including endogenous opiates, and a critical distinction has been made between the *wanting* that is produced by dopaminergic activity and the *liking* produced by the opioid system (Berridge, 2007). This distinction has been demonstrated extensively through pharmacological manipulation in rodents, but relevant human studies exist as well. For example, administering an opiate antagonist together with amphetamine eliminated the pleasure otherwise associated with amphetamine (Jayaram-Lindström et al., 2004).

Dopamine most purely seems to produce desire to seek reward (i.e., to achieve some goal) or to discover information. This desire is not necessarily pleasant. When working hard for a reward that is highly uncertain, for example, or when progress is frustratingly slow, the desire that is driven by dopamine may involve little pleasure in and of itself, and may even be experienced as unpleasant. This is true as well of the desire for information associated with the salience system. People sometimes describe themselves as “dying of curiosity” or “dying” to reach a particular goal—it is safe to assume that the use of “dying” as a metaphor rarely signals straightforward enjoyment. To be extremely eager can be emotionally painful. Of course, the desire for specific rewards or information can be accompanied by intense pleasure when progress toward the goal is satisfactory (cf. Carver and Scheier, 1998), but that particular type of pleasure is likely to be due to the combination of dopamine release by the value coding system with release of endogenous opiates.

The role of the opioid system in pleasure does not mean that high-arousal pleasure states like elation and excitement should not be considered dopaminergic emotions, because they are probably never experienced due to opioid activity alone but rather require dopaminergic activity as well. (Opiate related pleasure without dopaminergic activity is likely to be experienced as a more relaxed pleasure, involving satisfaction or bliss, rather than elation and excitement.) However, the importance of the opioid system for pleasure does highlight the fact that dopaminergic emotions are not simply pleasant and that they reflect wanting more specifically than liking. They are likely to include a variety of emotions oriented toward future acquisition of reward or information: desire, determination, eagerness, interest, excitement, hope, curiosity (cf. Silvia, 2008). (This list is not intended to be exhaustive.) At present, we can only speculate about the difference between emotions associated specifically with the value system vs. the salience system. Emotions related to specific rewards, like elation or craving, seem likely to be driven primarily by the value system, whereas curiosity seems likely to be driven primarily by the salience system. Surprise seems likely to be an emotion tied to the alerting signal (Bromberg-Martin et al., 2010). The full range of emotions related to dopamine should be a fruitful topic for future research.

#### Involuntary versus voluntary encounter with the unknown

Up to this point, increases in psychological entropy have been described primarily as the result of stimuli to which individuals are involuntarily exposed. This framing glosses over one of the most important facts about exploration, namely that it frequently entails voluntary efforts to increase psychological entropy, to put oneself in situations where one is uncertain of what to do or how to understand what is happening. This is a relatively straightforward consequence of the fact that uncertainty has innate incentive reward value, but its implications must not be overlooked. People seek incentive rewards just as they seek consummatory rewards; thus, people are motivated to seek increases in psychological entropy. Individual differences in dopaminergic function influence not only what people do when confronted with the unknown but also the degree to which they will eagerly seek out the unknown. Individual differences in exploration are evident in everything from mountain climbing to reading. Why there is some value in exploring in the presence of anomaly is obvious. What is more complicated is why there is value in unprompted exploration, the creation of additional psychological entropy even when no threat to any particular goal is evident.

A mechanism that supplies psychological entropy with reward value not only serves to encourage learning when anomaly is encountered, it also drives the organism to look for anomaly even when this is not necessary. From an evolutionary perspective, unnecessary exploration may be advantageous, despite attendant risk, because it tends to increase potentially useful knowledge about the environment, which may sooner or later facilitate either acquisition of reward or avoidance of punishment. EMU posits the evolutionary function of voluntary exploration to be a long-term decrease in entropy—that is, a more effective strategy for pursuing the goals of the organism (Hirsh et al., 2012), and my extension of EMU does not alter that assumption. However, evolution does not need to instantiate a particular goal directly, as long as the goals it does instantiate serve that function; for example, evolution does not need to instill a desire for offspring as long as it instills a desire for sex. Because of the innate incentive value of uncertainty, people desire exploration for its own sake (i.e., they treat it as a goal in itself) and engage in it even at times when exploration will not obviously further their goals. The exploration theory of dopamine posits that, although human beings are indeed “motivated to reduce the experience of uncertainty to a manageable level” (Hirsh et al., 2012, p 4), they are also motivated to increase the experience of uncertainty to an interesting level—in other words, to a level at which some previously unknown reward or information may be discovered. Thus, exploration is used not only to transform the unknown into the known, but also the known into the unknown (Peterson, 1999). The value system seems likely to drive unprompted, but potentially fruitful, behavioral exploration of the social and physical world, whereas the salience system seems likely to drive spontaneous innovation and cognitive exploration.

## Dopamine and personality

With a working model of the role of dopamine in the human cybernetic system, we can now turn to personality. How do individual differences in the functioning of the dopaminergic system relate to individual differences in personality traits? Personality traits are probabilistic descriptions of the frequency and intensity with which individuals exhibit particular behavioral, motivational, emotional, and cognitive states (Fleeson, 2001; Fleeson and Gallagher, 2009; DeYoung, 2010b; Corr et al., 2013). The major goal of personality neuroscience is to identify the mechanisms that produce those states and the parameters of those mechanisms that vary to influence personality traits (DeYoung, 2010b). In the previous sections, I have elaborated on the exploratory states that are associated with dopaminergic function. In what follows, I develop a theory of the traits related to those states.

Three broad dopaminergic parameters seem likely to be centrally important for determining personality traits: (1) global levels of dopamine, determined by genetic and metabolic processes that influence availability of dopamine throughout the dopaminergic system, (2) level of activity in the value coding dopaminergic system, and (3) level of activity in the salience coding dopaminergic system. Obviously, some individual differences in behavior and experience are likely to be associated with additional parameters more fine-grained than these three, such as the density of different dopaminergic receptors in different brain structures, or the efficiency of different mechanisms of synaptic dopamine clearance. Nonetheless, the extent of available evidence is not yet conducive to compelling theory at that level of detail, and I will only occasionally speculate about such effects, when it is particularly relevant to the evidence in question.

An important premise in many theories of the biological basis of personality is that traits reflect relatively stable responses to broad classes of stimuli (Gray, 1982; Corr et al., 2013). (Note that this should alleviate any concern that personality trait constructs are inadequate to describe human behavior because they are not context sensitive. They are indeed context sensitive, but the broader the class of stimuli in question, the more contexts to which they will be relevant.) With this in mind, we can identify uncertain or unpredicted stimuli as the very broad class to which all traits influenced by dopamine are responses. Other traits (e.g., Neuroticism) may also reflect stable patterns of response to uncertainty, but they reflect different types of response (aversive or defensive responses in the case of Neuroticism). Dopaminergic traits reflect individual differences in incentive responses to uncertainty. Global level of dopamine should influence typical exploratory responses to the incentive value of all kinds of uncertainty. Activity level in the value system should influence typical exploratory responses to cues of specific reward, and activity level in the salience system should influence typical exploratory responses to cues of information.

### Personality structure: dopamine in the big five hierarchy

The core of the present theory is that activity level in the value system is reflected in *Extraversion*, activity level in the salience system is reflected in *Openness/Intellect*, and global levels of dopamine are reflected in the metatrait *Plasticity*, which represents the shared variance of Extraversion and Openness/Intellect (DeYoung, 2006). All other traits influenced by dopamine are hypothesized to be related to these three traits or one of their subtraits (although not every trait related to these three traits is presumed to be influenced by dopamine). To understand why these are the primary traits of interest requires some discussion of personality structure. The goal of the present theory is to link a theory of dopamine to what is already known about the structure of personality in general. One might instead ignore the history of research on personality structure and posit a trait of exploration, or interest, or curiosity, or engagement, and then develop a questionnaire scale specifically targeting that trait (e.g., Kashdan et al., 2004). Indeed, if the present theory is correct, such a scale would be likely to correspond well to the trait manifestation of dopaminergic function in personality, but, additionally, it should be very strongly related to Plasticity, due to the comprehensiveness of the Big Five as a taxonomy.

Extraversion and Openness/Intellect are two of the Big Five personality traits, which also include Conscientiousness, Agreeableness, and Neuroticism (John et al., 2008). The Big Five system (also known as the Five-Factor Model) was developed empirically, through factor analysis of patterns of covariance among ratings of personality using trait-descriptive adjectives taken from the lexicon (Goldberg, 1990). Very similar five-factor solutions have been found in many languages^4^. Importantly, the Big Five appear not only in lexical research, but also in factor analysis of many existing personality questionnaires, even when those questionnaires were not designed to measure the Big Five (Markon et al., 2005). Additionally, factors closely resembling the Big Five appear in factor analysis of symptoms of personality disorder (Krueger et al., 2012; De Fruyt et al., 2013).

The major premise of the Big Five as a taxonomy is that the same five latent factors are present in any sufficiently comprehensive collection of personality assessments. This means that five major dimensions underlie most variation in human personality, and personality neuroscience should focus on explaining the mechanisms and parameters that are responsible for the coherence of these dimensions. Extraversion, for example, represents the shared variance of diverse traits including gregariousness, assertiveness, positive emotionality, and excitement seeking. Personality neuroscience needs to explain what these traits have in common in their underlying neurobiological processes. Given that the brain controls all behavior, personality traits must proximally be produced by variation in brain function, regardless of their distal sources in genetic and environmental influences (DeYoung, 2010b). Because the brain is a single unified cybernetic system, biological theories for all specific traits should be compatible and ultimately unified. Thus, theories of specific, theoretically-derived personality traits (e.g., exploration or curiosity) should not stand alone, but should rather be integrated with theories based on the Big Five.

**Figure 1:**
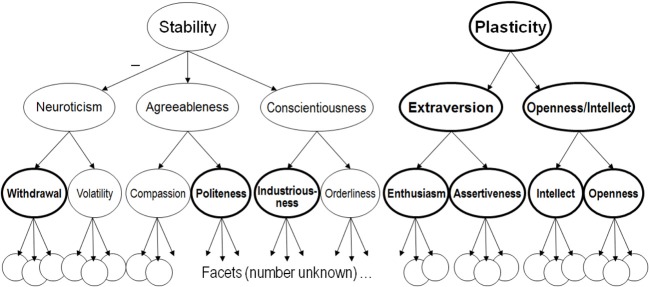
**The Big Five personality trait hierarchy (DeYoung, 2006, 2010b; DeYoung et al., 2007).** Traits outlined in bold are hypothesized to be influenced by dopamine.

The other crucial fact about personality structure for the present theory is that traits are organized hierarchically (Figure 1). Traits near the top of the personality hierarchy represent broad regularities in psychological functioning, encompassing many different types of behavior and experience that tend to vary together. Narrower traits lower down in the hierarchy represent more limited sets of behavior and experience that tend to vary together. Important traits exist both above and below the Big Five in the personality hierarchy (Markon et al., 2005; DeYoung, 2006; DeYoung et al., 2007). Although the Big Five were originally assumed to be orthogonal and the highest level of the personality hierarchy, they have been demonstrated to have a regular pattern of intercorrelation that reveals the existence of two higher-order personality factors (Digman, 1997; DeYoung, 2006; Chang et al., 2012), and these higher-order factors or *metatraits* are also evident in genetic correlations derived from samples of twins (McCrae et al., 2008). We labeled the metatraits *Stability* (the shared variance of Conscientiousness, Agreeableness, and reversed Neuroticism) and *Plasticity* and hypothesized that they reflect the primary manifestations in personality of individual differences in serotonergic and dopaminergic function, respectively (DeYoung et al., 2002; DeYoung and Gray, 2009).

Below the Big Five in the personality trait hierarchy are two additional levels of structure. The bottom level of the hierarchy is described as containing *facets*, many narrow traits that form the constituent elements of all broader dimensions. No consensus exists as to the number and identity of the facets, and different instruments assess different collections of facets. Recently, a level of personality structure has been discovered between the many facets and the Big Five domains, appearing first in behavioral genetic research in twins, which found that two genetic factors were necessary to explain the covariance among the six facets in each Big Five domain as measured by the popular NEO Personality Inventory-Revised (NEO PI-R; Costa and McCrae, 1992b; Jang et al., 2002). If the Big Five were the next level of the personality hierarchy above the facets, only one genetic factor would be necessary for each domain. This finding was extended by a non-genetic factor analysis of 15 facet scales within each Big Five domain that found evidence for the existence of exactly two factors in each of the Big Five (DeYoung et al., 2007). These factors corresponded sufficiently closely to the previously reported genetic factors to suggest that both studies might be describing the same intermediate level of structure within the Big Five hierarchy. Traits at this level were described as *aspects*, with each of the Big Five having two aspects, and the aspect factors were characterized by correlating them with over 2000 items from the International Personality Item Pool. This procedure enabled the construction of an instrument to measure the aspects, the Big Five Aspect Scales (BFAS; DeYoung et al., 2007).

The aspect level of personality structure is important in part because it is empirically derived, whereas most lists of facets have been rationally derived. The 10 aspects of the Big Five provide a less arbitrary system than the facets for investigating personality traits below the Big Five, and they seem likely to represent the most important differentiations for discriminant validity within each of the Big Five (e.g., DeYoung et al., 2013a). As well as discussing evidence for the relation of dopamine to Extraversion, Openness/Intellect, and Plasticity, I argue that the aspect-level of the personality hierarchy is important for understanding the full extent of dopamine's influence on personality, as depicted in Figure 1. Crucially, traits at lower-levels of the hierarchy contain unique genetic variance, not shared with traits at higher levels (Jang et al., 2002). Thus, dopamine may influence aspect level traits without influencing the traits above them in the hierarchy.

### Extraversion

The dimension identified as Extraversion in the Big Five represents the shared variance among traits including talkativeness, sociability, leadership, dominance, activity level, positive emotionality, and excitement seeking. The various facets of Extraversion group into two related but separable aspects, *Assertiveness* and *Enthusiasm*, with Assertiveness encompassing traits like leadership, dominance, and persuasiveness, and Enthusiasm encompassing sociability or gregariousness and positive emotionality. Some traits, like talkativeness, are shared by both Assertiveness and Enthusiasm. One facet of Extraversion that does not fit neatly into either major aspect of the trait is excitement seeking, which will be discussed in the section *Impulsivity and Sensation Seeking* with related constructs like sensation seeking and novelty seeking (DeYoung et al., 2007; Quilty et al., 2013).

Extraversion is the trait most commonly linked to dopamine in the existing personality literature, and Extraversion is believed to reflect the primary manifestation in personality of sensitivity to reward (Depue and Collins, 1999; Lucas and Baird, 2004; Smillie, 2013). A number of studies have found evidence of a link between Extraversion and dopamine using pharmacological manipulation of the dopaminergic system (Depue et al., 1994; Rammsayer, 1998; Wacker and Stemmler, 2006; Wacker et al., 2006, 2013; Depue and Fu, 2013). Although Extraversion is often viewed as a social trait, it encompasses more than just social behavior, including physical activity level and positive emotion even in non-social situations. Further, its social component can be seen as the direct result of the fact that many human rewards are social; among the most potent human rewards are social status or dominance and interpersonal affiliation. Sensitivity to the reward value of status appears to be associated primarily with Assertiveness, whereas sensitivity to the reward value of affiliation appears to be associated primarily with Enthusiasm (DeYoung et al., 2013a).

**Figure 2:**
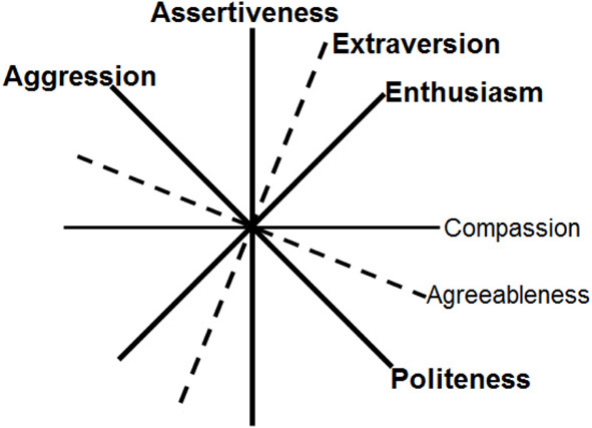
**Angular relations among the aspects of Extraversion and Agreeableness correspond to the interpersonal circumplex (DeYoung et al., 2013a).** Aggression characterizes the low pole of Politeness. Traits in bold are hypothesized to be influenced by dopamine.

In a similar vein, Depue and colleagues (Depue and Collins, 1999; Depue and Morrone-Strupinsky, 2005) have distinguished between *Agentic Extraversion* and *Affiliative Extraversion*, which correspond reasonably well to Assertiveness and Enthusiasm, respectively. However, they have tended to lump traits related to Agreeableness together with Affiliative Extraversion, which can be misleading because Enthusiasm appears to entail finding affiliation rewarding, whereas Agreeableness appears to be related to affiliation for other reasons (such as the ability to empathize). Agreeableness reflects differences in the various forms of altruistic social behavior. The relations among Extraversion and Agreeableness can be clarified by noting that these two traits define the interpersonal circumplex (IPC), a two-dimensional model widely used to describe social behavior (DeYoung et al., 2013a). The two aspects of Agreeableness are Compassion, describing empathy and concern for the feelings and desires of others, and Politeness, describing suppression of rude or aggressive behavior. Assertiveness and Compassion correspond to the vertical and horizontal axes of the IPC, and Enthusiasm and Politeness correspond to the diagonal axes at 45 and 315° (Figure 2). Because Enthusiasm and Compassion are adjacent axes of the circumplex, they are as strongly correlated with each other as with the other aspect of their respective Big Five trait, and this has led some researchers to blur the distinction between Compassion and Enthusiasm. Such blurring is likely to be problematic for personality neuroscience, given the hypothesis that Enthusiasm is related to reward sensitivity but Compassion is not (DeYoung et al., 2013a).

In previous work, we have hypothesized that Assertiveness and Enthusiasm reflect *wanting* and *liking* respectively, which would suggest that only Assertiveness should be directly related to dopaminergic function (DeYoung, 2010b; Corr et al., 2013; DeYoung et al., 2013a). This would be consistent with the hypothesis of Depue and Collins (1999) that Agentic Extraversion, specifically, is related to dopamine. This contrast is probably overly simplistic, however. Based on the emotional content associated with Enthusiasm and a study by Smillie et al. (2013), the current theory proposes that Enthusiasm reflects a combination of wanting and liking, whereas Assertiveness is a purer reflection of wanting. The most explicitly emotional items in the BFAS assessment of Enthusiasm are, “Rarely get caught up in the excitement,” “Am not a very enthusiastic person,” and “Show my feelings when I'm happy” (DeYoung et al., 2007). These are the sort of eager, vigorous emotional responses that suggest dopaminergic activation in response to the promise or delivery of reward. Of course, they are also suggestive of hedonic pleasure in the receipt or imagination of reward, and the present theory maintains the hypothesis that variance in Enthusiasm reflects variation in the opioid system but proposes that it is also influenced by the dopaminergic value system. This would be consistent with the finding that both Assertiveness and Enthusiasm similarly predicted high levels of activated positive affect (e.g., feeling “energetic” and “active”) in response to an appetitive film clip depicting vigorous goal-directed behavior (Smillie et al., 2013). These findings suggest that both Assertiveness and Enthusiasm predict individual differences in emotional response to the kind of incentive cues that trigger dopaminergic activity in the value system. Nonetheless, because Enthusiasm is assumed to reflect liking as well as wanting, variance in Assertiveness is hypothesized to be more strongly related to dopamine than is variance in Enthusiasm (cf. Wacker et al., 2012).

No discussion of the relation of Extraversion to dopamine could be complete without reference to the work of Jeffrey Gray, who was one of the first researchers to develop a biological personality model based on the premise that traits represent consistent individual differences in responses to different classes of stimuli (Gray, 1982). Gray developed a “conceptual nervous system” that included a Behavioral Activation or Approach System (BAS) to respond to cues of reward and a Behavioral Inhibition System (BIS) and Fight-Flight-Freeze System (FFFS) to respond to threats (Gray and McNaughton, 2000). Personality traits are proposed to result from individual difference in the sensitivity of these systems. The biological basis of the BAS was never fleshed out as thoroughly as that of the BIS and FFFS, but its core was always presumed to be the dopaminergic system and its projections to the striatum (Pickering and Gray, 1999). Panksepp (1998) has posited a similar system centered around dopaminergic function, which he labeled the SEEKING system.

Gray (1982) originally considered the trait associated with BAS sensitivity to be different from Extraversion and suggested that it could be characterized as *Impulsivity*. More recent research, however, suggests that measures of BAS sensitivity assess the same latent trait as measures of Extraversion and that impulsivity is a distinct trait (Zelenski and Larsen, 1999; Elliot and Thrash, 2002; Pickering, 2004; Smillie et al., 2006; Wacker et al., 2012). One of the most popular measures of BAS sensitivity includes three subscales, Drive, Reward Sensitivity, and Fun Seeking (Carver and White, 1994). Drive appears to be a reasonably good indicator of Assertiveness, whereas Reward Sensitivity may be more related to Enthusiasm (Quilty et al., 2013), although one study found that it loaded with Drive on an Agentic Extraversion factor (Wacker et al., 2012). Fun Seeking is similar to Excitement Seeking and will be discussed below in the section *Impulsivity and Sensation Seeking*. Total BAS sensitivity scores from this instrument have been shown to predict pharmacological responses to a dopaminergic drug (Wacker et al., 2013).

If Extraversion is the primary manifestation of reward sensitivity in personality, a major contributor to that sensitivity seems likely to be the tendency to seek and learn about possible rewards, which is driven by the value coding dopaminergic system. Most of the behaviors associated with Extraversion function as forms of exploratory behavior designed to pursue rewards. (Note that speech is an important mode of behavior in social interactions, often used to pursue rewards related to status and affiliation.) Extraversion has been shown to predict better learning under conditions of reward in reinforcement learning paradigms (Pickering, 2004; Smillie, 2013), as well as to predict facilitation of reaction times and accuracy following rewarding stimuli (Robinson et al., 2010). A recent study showed that Extraversion predicted the tendency for Pavlovian conditioning to take place when subjects were given a dopamine agonist rather than a placebo (Depue and Fu, 2013).

In addition to the pharmacological studies of dopamine mentioned above, neuroimaging studies provide evidence of the link between Extraversion and the brain systems involved in reward. Several structural MRI studies have found that Extraversion is associated with greater volume of VMPFC, a region known to be innervated by the value coding dopaminergic system and involved in coding the value of rewards (Omura et al., 2005; Rauch et al., 2005; DeYoung et al., 2010; but see Kapogiannis et al., 2012, for a failure to replicate). A few fMRI studies have shown that brain activity in response to monetary rewards or pleasant emotional stimuli is associated with Extraversion, but their samples sizes have typically been very small (*N* < 20), rendering their findings inconclusive (Canli et al., 2001, 2002; Cohen et al., 2005; Mobbs et al., 2005). Nonetheless, on the whole, a compelling body of evidence suggests that Extraversion may reflect the primary manifestation of individual differences in the value coding dopaminergic system as it interacts with other elements of the brain's reward systems. Extraversion has been described in a cybernetic context as an energizer of behavior (Van Egeren, 2009), precisely the role ascribed to tonic levels of dopamine (Niv et al., 2007). This description is congruent with the present theory, as long one specifies that it is exploratory behavior specifically that is energized by dopamine, and that behavior energized by the value coding system corresponds primarily to Extraversion, whereas behavior energized by the salience system corresponds primarily to Openness/Intellect.

### Openness/intellect

Openness/Intellect describes the general tendency to be imaginative, curious, perceptive, creative, artistic, thoughtful, and intellectual. The psychological process unifying these traits has been identified as “cognitive exploration,” with cognition conceived broadly to include both reasoning and perceptual processes (DeYoung et al., 2012; DeYoung, in press)^5^. The trait's compound label stems from an old debate, with some researchers favoring “Openness to Experience” and others “Intellect” (e.g., Goldberg, 1990; Costa and McCrae, 1992a). In fact, these two labels capture the two distinct (but equally important) aspects of the trait, with Intellect reflecting engagement with abstract information and ideas and Openness reflecting engagement with perceptual and sensory information (Saucier, 1992; Johnson, 1994; DeYoung et al., 2007). When I refer to “Openness/Intellect,” I am referring to the Big Five dimension; when I refer to either “Intellect” or “Openness” alone, I am referring just to one subtrait within Openness/Intellect. Traits within Intellect include intelligence, perceived intelligence or intellectual confidence, and intellectual engagement, whereas traits within Openness include artistic and aesthetic interests, absorption in sensory experience, fantasy proneness, and apophenia or overinclusive pattern detection (DeYoung et al., 2012; DeYoung, in press). (The inclusion of intelligence within Intellect is controversial and will be discussed further below.) The present theory posits that variation in Openness/Intellect reflects, in part, variation in the salience coding dopaminergic system.

The evidence for involvement of dopamine in Openness/Intellect is more circumstantial than the evidence for Extraversion, with the exception of two molecular genetic studies showing associations with the *DRD4* (dopamine D4 receptor) and *COMT* genes in three samples (Harris et al., 2005; DeYoung et al., 2011). COMT (catechol-*O*-methyltransferase) is an enzyme that degrades dopamine and is important for synaptic clearance. Because D4 receptors are localized primarily in the cortex (Meador-Woodruff et al., 1996; Lahti et al., 1998), and because COMT is believed to be more influential on dopaminergic levels in the cortex than in the striatum (Tunbridge et al., 2006), these associations seem particularly likely to be related to cognitive exploration and the salience coding dopaminergic system. Nonetheless, molecular genetic studies are notoriously difficult to replicate, and the circumstantial evidence is, therefore, additionally important.

We originally hypothesized that dopamine is involved in the biological substrate of Openness/Intellect based on four lines of evidence (DeYoung et al., 2002, 2005). First, as noted above, the involvement of dopamine in curiosity and exploratory behavior is well-established. Given the centrality of curiosity to the Openness/Intellect factor, and its relation to exploratory traits like novelty seeking and sensation seeking (Costa and McCrae, 1992a; Aluja et al., 2003), the conceptual link to dopamine is obvious. Second, dopamine is involved in the mechanisms that support cognitive exploration specifically, being necessary for working memory function and also contributing to learning. Openness/Intellect is the only Big Five trait positively associated with working memory ability, and its Intellect aspect has been shown to predict neural activity in the PFC that is correlated with working memory performance (DeYoung et al., 2005, 2009). These findings suggest that variations in salience coding dopaminergic function in PFC might be partly responsible for the cognitive attributes associated with Openness/Intellect. Third, Openness/Intellect appears to be associated with reduced latent inhibition (Peterson and Carson, 2000; Peterson et al., 2002). Latent inhibition is an automatic pre-conscious process that blocks stimuli previously categorized as irrelevant from entering awareness. Dopamine appears to be the primary neuromodulator of latent inhibition, with increased dopaminergic activity producing reduced latent inhibition (Kumari et al., 1999). Finally, the correlation of Openness/Intellect with Extraversion, which reveals the metatrait Plasticity, is itself suggestive that dopamine may be one cause of their covariance, given the evidence for dopamine's involvement in Extraversion.

Highlighting the fact that the division of the dopaminergic system into salience and value coding systems is coarse, and that each system has multiple subcomponents, the salience coding dopaminergic system seems likely to play somewhat different roles in Intellect vs. Openness. Intellect rather than Openness is uniquely associated with general intelligence and working memory (DeYoung et al., 2009, 2013b; Kaufman et al., 2010) and seems likely to reflect dopamine's facilitation both of voluntary reasoning processes that rely on DLPFC and of motivation to reason about experience. Openness, in contrast, appears likely to reflect dopamine's facilitation of the detection of patterns in sensory experience (Wilkinson and Jahanshahi, 2007). One study found a double dissociation in which Intellect predicted working memory, but Openness predicted implicit learning, the automatic detection of patterns (Kaufman et al., 2010). Implicit pattern detection is likely to be modulated by dopamine's action in the striatum rather than the prefrontal cortex, and different branches of the salience system project to these two brain regions. Additionally, Openness may be particularly influenced by dopaminergic projections to the thalamus, which are likely to play an important role in controlling the flow of sensory information to the cortex and basal ganglia (Sánchez-González et al., 2005). Finally, Openness, like Enthusiasm, seems likely to be influenced by the opioid system as well as by dopamine, because aesthetic pleasure (the enjoyment of sensory patterns) is one of its key features (DeYoung, in press). On the whole, Intellect seems likely to be more strongly linked to dopamine than Openness.

#### Intelligence

The inclusion of intelligence within Intellect is controversial. I have made the case for it elsewhere (DeYoung, 2011, in press; DeYoung et al., 2012) and will not reiterate all the arguments here because, for the present theory, it is irrelevant whether one considers intelligence to be a facet of Intellect or a separate but related trait. In either case, the pattern is maintained that all traits influenced by variation in dopaminergic function are related to Plasticity and/or its subtraits. Intelligence has traditionally been separated from most personality traits by its method of assessment, performance tests as opposed to questionnaires. Intelligence scores are therefore more specifically an index of ability than are any scores derived from questionnaires. Nonetheless, integrating intelligence mechanistically with the rest of personality is important to further the development of a coherent neurobiological explanation of individual differences. Because the brain is a single system of interacting elements, mechanistic theories for all specific traits should be compatible and ultimately unified. One of the mechanisms that may link intellectual confidence and engagement with intellectual ability or intelligence is the function of the salience system as it facilitates working memory and explicit learning. Considerable evidence implicates working memory capacity as one of the major contributors to general intelligence (Conway et al., 2003; Gray et al., 2003), although other factors, like processing speed, and the ability to learn associations voluntarily are likely to contribute as well (Kaufman et al., 2009). Given the importance of dopamine for working memory, dopamine's link to intelligence is highly likely.

Nonetheless, the evidence directly linking dopamine to tests of intelligence is not extensive. Some of the best evidence comes from research on cognitive aging, which has been associated with the variation in the normative decline in dopamine with age. Even controlling for age, dopaminergic function assessed by positron emission tomography (PET) has been found to predict intelligence in these studies (Volkow et al., 1998; Erixon-Lindroth et al., 2005). Different components of the salience system may influence intelligence differently, with binding at D1-type receptors facilitating reasoning and binding at D2-type receptors facilitating cognitive flexibility (Wacker et al., 2012).

#### Creativity

Whereas the inclusion of intelligence within the general Openness/Intellect factor is controversial, the inclusion of creativity is not. The general tendency toward innovation, originality, and creativity is common to both aspects of the trait and is the facet most central to Openness/Intellect as a whole (Johnson, 1994; DeYoung, in press). Indeed, Johnson (1994) proposed *Creativity* as an alternative label for the Openness/Intellect factor. This proposal was based primarily on the relation of various trait-descriptive adjectives to the Openness/Intellect factor, but it has been amply demonstrated that Openness/Intellect is the best Big Five predictor of creativity, whether creativity is measured through performance tests in the lab or by creative achievement in real life (McCrae, 1987; Feist, 1998; Carson et al., 2005; Chamorro-Premuzic and Reichenbacher, 2008). Creativity is typically defined as the ability to generate products (abstract or material) that are simultaneously novel and useful or appropriate (Mumford, 2003; Simonton, 2008).

Creative achievement, like Openness/Intellect, is associated with reduced latent inhibition, which presumably allows the creative person to perceive possibilities that others would automatically ignore and suggests the importance of dopamine for creativity (Carson et al., 2003). More directly, both genetic and neuroimaging studies have linked dopamine to performance on creativity tests (Reuter et al., 2006; de Manzano et al., 2010). Finally, multiple studies have found that creative performance is predicted by eye-blink rate, which is a marker of dopaminergic activity that also predicts Extraversion (Depue et al., 1994; Chermahini and Hommel, 2010, 2012).

#### Positive schizotypy or apophenia

Schizotypy is a personality trait (more precisely, a cluster of traits) that reflects subclinical levels of symptoms of schizophrenia-spectrum disorders in the general population, and it is a major liability factor for those disorders. Dopamine has long been implicated in schizophrenia, and most anti-psychotic medications are dopamine antagonists. Importantly, excess dopamine seems to be involved specifically in the psychotic, or *positive*, symptoms of schizophrenia, which include magical ideation, perceptual aberrations (e.g., hallucination), and overinclusive thinking (Howes et al., 2009, 2011). All the symptoms of positive schizotypy can be described as *apophenia*, the tendency to perceive meaningful patterns and causal connections where none in fact exist, and these symptoms are predicted by Openness (DeYoung et al., 2012; Chmielewski et al., in press). The tendency to detect covariance patterns, which is associated with Openness as well as apophenia (Kaufman et al., 2010), may lead to over-interpretation of coincidences and sensory noise as meaningful patterns. Indeed, apophenia as a trait is positively correlated with identification of meaningful patterns in noisy or random visual stimuli (Brugger et al., 1993; Blackmore and Moore, 1994). Apophenia may be caused, at least in part, by the low levels of latent inhibition that have been demonstrated repeatedly in psychosis and schizotypy (Lubow and Gewirtz, 1995; Gray et al., 2002). (Occasional failures to detect associations of latent inhibition with schizotypy may be due to the confounding of positive and negative symptoms. The latter comprise anhedonia—that is, lack of pleasure in sensory and social experience—and may actually be positively related to LI (Cohen et al., 2004), which is consistent with the association of anhedonia with dopamine, per the section *Depression and Anxiety* below.) In neuroimaging studies, schizotypy has predicted D2 receptor density and dopamine release in response to amphetamine (Woodward et al., 2011; Chen et al., 2012). Excess dopamine has been described as producing “aberrant salience” in schizophrenia-spectrum disorders (Kapur, 2003). The association of apophenia with Openness suggests that both may be influenced by level of activity in the salience system (DeYoung et al., 2012), although apophenia seems likely to be more specifically related to dopamine than is Openness more generally.

Inclusion of positive schizotypy or apophenia as a facet of Openness is nearly as controversial as inclusion of intelligence as a facet of Intellect, in part because apophenia is weakly negatively correlated with intelligence and nearly uncorrelated with questionnaire measures of Intellect. Nonetheless, we have shown that both apophenia and intelligence load positively on the general Openness/Intellect factor, and that when Openness and Intellect are separated, then apophenia loads strongly with Openness (DeYoung et al., 2012). The negative association of apophenia with intelligence suggests it could be caused in part by an imbalance of dopaminergic function in different branches of the salience system. If striatal dopamine is highly active in response to salient events, encouraging the assignment of meaning to correlational patterns, but dopamine levels in DLPFC are either too high or too low to support working memory and intelligence, this could lead to difficulty differentiating likely from unlikely patterns (cf. Howes and Kapur, 2009). (Of course, deficits in intelligence with causes entirely unrelated to dopamine could also produce apophenia in conjunction with high levels of activity in the salience coding system.) Apophenia is clearly linked to Openness and can be well-described as “openness to implausible patterns” (DeYoung et al., 2012).

In the Personality Inventory for the DSM 5 (PID-5; Krueger et al., 2012) and in the Personality Psychopathology Five model (PSY-5; Harkness et al., 1995), positive schizotypy or apophenia is labeled *Psychoticism*. The construct measured by the PID-5 and other scales assessing apophenia should not be confused with the construct measured by Eysenck's Psychoticism scale, which most personality psychologists agree was mislabeled, as it measures antisocial and impulsive behavior (sometimes called “impulsive non-conformity”) rather than positive schizotypy (Goldberg and Rosolack, 1994; Pickering, 2004; Zuckerman, 2005). Some have considered impulsive non-conformity to be a facet of schizotypy, but it is distinct from the positive psychotic symptoms that are characterized by apophenia. Eysenck's Psychoticism does not appear to predict risk for schizophrenia diagnosis (Chapman et al., 1994; Vollema and van den Bosch, 1995). Studies linking Eysenck's Psychoticism to dopamine (e.g., Kumari et al., 1999) are thus most relevant to the sections *Impulsivity and Sensation Seeking* and *Aggression* below, which discuss impulsivity and aggression.

### Plasticity

Plasticity, the shared variance of Extraversion and Openness/Intellect, in a sense forms the core of the present theory. This very broad trait should be influenced by forces that alter global dopaminergic tone and thus increase or decrease activity of both the value and salience systems. For now the only evidence for this hypothesis is the evidence, described above that dopamine is involved in both Extraversion and Openness/Intellect. In future, the hypothesis that Plasticity should predict global levels of dopamine may be tested directly.

The label “Plasticity” has the potential to be confusing because the term is more often applied to brain function than to personality. Psychologists are probably most familiar with it in the context of the phrase “neural plasticity,” which refers to the ability of the brain to alter many aspects of its neural architecture in response to experience. *Plasticity*, as a personality trait, is not intended to be synonymous with “neural plasticity,” regardless of the degree to which neural plasticity plays a role in the exploratory processes associated with Plasticity. Similarly, *Stability*, as a personality trait, is not synonymous with “neural stability.” Rather, the terms refer to the stability and plasticity of the cybernetic elements that constitute the individual psychologically (DeYoung, 2010c). Recall that the cybernetic system encompasses (1) desired end states or goals, (2) knowledge and evaluations of the current state, and (3) operators potentially capable of transforming the current state into the goal state. As a parameter of this system, the metatrait Stability is hypothesized to reflect the degree to which the individual resists disruption of ongoing goal-directed functioning by distracting impulses, maintaining stable goal-representations and relevant evaluations of the present, and selecting appropriate operators^6^. Plasticity is hypothesized to reflect the degree to which the cybernetic system is prone to generating new goals, new interpretations of the present state, and new strategies to pursue existing goals (this is a description of exploration in cybernetic terms). As personality traits, Stability and Plasticity reflect between-person variation in the processes that fulfill two basic needs of any cybernetic system in an environment that is not fully predictable: first, to be able to maintain the stability of its own functioning so that goals may be accomplished, and second, to be able to explore complex, changing, and unpredictable circumstances, thereby increasing the adaptive effectiveness of its goal pursuit.

Stability and Plasticity may seem conceptually opposed, but it would be more accurate to describe them as in tension. Of course, heightened Plasticity may make Stability a challenge, but without adequate adaptation enabled by Plasticity, the individual will not long remain stable in an unpredictably changing environment. Because of the nested nature of subgoals within goals, processes associated with Plasticity can generate new subgoals in the service of a higher-order goal that is being maintained by processes associated with Stability. Further, without adequate Stability, the magnitude of psychological entropy is likely to be great enough that aversion wins out over exploration, leading to reduced Plasticity. When the Big Five are measured using ratings from multiple informants, Stability and Plasticity appear to be uncorrelated (DeYoung, 2006; Chang et al., 2012). The opposite of “stability” is “instability” not “plasticity,” and the opposite of “plasticity” is “rigidity” or “inflexibility” rather than “stability.” A well-functioning cybernetic system must be both stable and plastic.

In short, the function associated with Plasticity is posited to be precisely that which dopamine facilitates: to explore and thus to achieve the rewards inherent in the positive potential of uncertainty. Several studies have supported predictions based on this theory. (For an effect to be considered associated with Plasticity, it should be associated with both Extraversion and Openness/Intellect with roughly similar magnitude, so that it is truly their shared variance driving the effect, rather than variance at the Big Five level.) For example, Plasticity was found to predict self-reported moral conformity negatively, based on the premise that those who conform to societal moral expectations are less likely to be exploratory or to rely on their own adaptive capacity (DeYoung et al., 2002). Plasticity was also found to positively predict Externalizing (a factor indicating the general tendency toward impulsivity, aggression, antisocial behavior, and drug use), following the premise that externalizing behavior is driven in part by motivation to explore behaviors that are socially unacceptable, and the fact (discussed below) that externalizing behaviors have been associated with dopamine (DeYoung et al., 2008). Stability also predicted conformity and Externalizing, in the opposite direction from Plasticity. In fact, Stability was the primary correlate of both of these characteristics, and the association with Plasticity was not evident unless one controlled for Stability^7^.

It is particularly of interest to identify behaviors that are primarily associated with Plasticity rather than Stability. The general tendency to explore may not be most purely manifested in behaviors that are most strongly associated with common colloquial meanings of “exploration,” such as pursuing experiences that are extremely novel to the individual or unusual or novel in society as a whole. Such particularly dramatic forms of exploration, especially when not socially sanctioned, may be predicted not only by Plasticity, but also by low Stability, as implied by the studies of conformity and externalizing behavior mentioned above.

What then are the best specific markers of Plasticity in the general population? In one large, middle-aged, middle-class sample (DeYoung, 2010c), the personality items that specifically characterized Plasticity were dominated by content reflecting leadership, skill, and expressiveness in social situations (e.g., “Have a natural talent for influencing people,” “Have a colorful and dramatic way of talking about things”) with some additional items also clearly reflecting innovation and curiosity (e.g., “Am able to come up with new and different ideas,” “Look forward to the opportunity to learn and grow”). In the same sample, we examined how Plasticity and Stability uniquely predicted the self-reported frequency, over the past year, of 400 behaviors (Hirsh et al., 2009). We found that Plasticity was almost universally a positive predictor of behavioral frequency, consistent with dopamine's role as a motivational energizer, and the behaviors it most strongly predicted were an intriguing collection, which included planning a party, attending a public lecture, attending a city council meeting, giving a prepared talk or public recital, writing a love letter, going dancing, and making a new friend, among others. Here we see the manifestation of a general exploratory tendency among middle-aged, middle-class Americans. (In contrast, Stability was almost universally a negative predictor of behavioral frequency, with the strongest effects on various impulsive or disruptive behaviors.) In the present theory, all of these behaviors associated with Plasticity should be among those most facilitated by increasing dopaminergic activity in both the value and salience systems simultaneously.

It should be noted that other interpretations and labels have been offered for the factor we label Plasticity. Digman (1997), who discovered the metatraits, labeled them simply *Alpha* (Stability) and *Beta* (Plasticity) and proposed that the latter reflects a tendency toward personal growth. Olson (2005, p 1692) labeled the Plasticity factor *Engagement* and argued that it reflects “the extent to which individuals actively engage their inner and outer worlds.” Further, the metatraits of the Big Five resemble the two-factor solution that has been reported in lexical studies, in which the trait containing content from both Extraversion and Openness/Intellect has been labeled *Dynamism* (Saucier et al., 2013). All these interpretations seem compatible with each other. A general tendency toward exploration will lead to active engagement with novel and interesting phenomena and should produce behavior that others find dynamic and that is likely to lead to personal growth.

#### Lack of simple structure and the relation of plasticity to industriousness and achievement striving

In order to understand the full extent of the probable role of Plasticity and dopamine in personality, it is important to understand one additional thing about the personality trait hierarchy—namely that it is an over-simplification. If the personality hierarchy were exactly as schematically depicted in Figure 1, none of traits located under Stability would be related to any of the traits located under Plasticity. However, it has long been known that personality does not have simple structure, in which each variable loads on one and only one factor (Costa and McCrae, 1992b; Hofstee et al., 1992). Attempting to fit the model depicted in Figure 1 to data from the BFAS, using confirmatory factor analysis, will yield a poor fit because of cross-loadings at the aspect-level (e.g., Ashton et al., 2009). Many lower-level traits are related to more than one higher level trait, and this is true even across the two sides of the hierarchy defined by the metatraits. I have already alluded to one example in the section on Extraversion (also depicted in Figure 2): although Extraversion and Agreeableness are unrelated, their aspects are systematically related, such that Enthusiasm is positively related to Compassion, and Assertiveness is negatively related to Politeness. Examining the pattern of correlation among the 10 aspects of the Big Five, and their lack of simple structure, suggests two important points regarding Plasticity. First, the shared variance of Extraversion and Openness/Intellect (i.e., Plasticity) appears to be due primarily to the association of Assertiveness and Intellect. These two traits are correlated with each other at about *r* = 0.5, at least as strongly as they are with the other aspect of the Big Five trait to which each belongs (DeYoung et al., 2007). Openness is considerably more weakly associated with the two aspects of Extraversion, and Enthusiasm is considerably more weakly associated with both aspects of Openness/Intellect. Second, there are two other aspect-level traits that are strongly correlated with Assertiveness and Intellect, as well as with each other; these are the Industriousness aspect of Conscientiousness and the Withdrawal aspect of Neuroticism. The latter encompasses anxiety and depression and predicts the other traits negatively.

This cluster of traits has been detected in slightly different guises in previous personality research. First, these aspect-level traits are all related to the lexical Dynamism factor (Saucier et al., 2013). Second, an attempt to discredit the existence of the metatraits, using the BFAS, purported to show that the metatraits could be rendered unnecessary by allowing aspect traits to cross-load on other Big Five factors—in other words, by taking into account their lack of simple structure (Ashton et al., 2009). Interestingly, however, the pattern of cross-loadings created an “Extraversion” factor that had similarly strong loadings not only for Enthusiasm and Assertiveness, but also for Intellect, Industriousness, and Withdrawal. Clearly, this is no longer just an Extraversion factor but rather a broader trait. In essence, a metatrait resembling Plasticity was recreated directly from the covariance of the aspect-level scales. Finally, in the Multidimensional Personality Questionnaire (MPQ), an Achievement scale that is strongly related to Conscientiousness and Openness/Intellect in the Big Five is grouped with scales reflecting Extraversion in a higher-order *Agentic Positive Emotionality* factor (Markon et al., 2005; Tellegen and Waller, 2008). In previously unpublished analysis of the BFAS and the MPQ in the Eugene-Springfield community sample (ESCS; Goldberg, 1999; *N* = 445), the Achievement scale showed its strongest correlations with Industriousness (0.30), Assertiveness (0.32), and Intellect (0.35). (The Achievement Striving scale from the NEO PI-R shows a similar pattern of correlations with the BFAS in this sample, *r* = 0.56, 0.46, and 0.31, respectively—the stronger correlation with Industriousness is not surprising, as this Achievement Striving scale was engineered as a facet of Conscientiousness). Confidence, ambition, and agency seem to be at the core of manifestations of Plasticity, and they are related not only to Extraversion (particularly Assertiveness), but also to Intellect and Industriousness and to a lack of Withdrawal. (The link between Withdrawal and dopamine is discussed below in the section *Depression and Anxiety*) The present theory posits that all of these traits are influenced by dopamine.

If the shared variance of Assertiveness and Intellect represents what is most central to Plasticity, one can understand the relation of Industriousness to Plasticity as reflecting the contribution that dopaminergic drive, in both value and salience systems, makes to the motivation for sustained hard work and the accomplishment of tasks. As noted above, dopamine appears to be crucial for overcoming the cost of effort when deciding to initiate behavior aimed at reward, especially as the probability of attaining the reward declines (Treadway and Zald, 2013). Industriousness is primarily an aspect of Conscientiousness, which reflects the capacity for top-down effortful control over impulses and distractions and is probably determined largely by characteristics of the prefrontal cortex (DeYoung et al., 2010), but Industriousness appears to have an important secondary contribution from Plasticity. To the extent that Industriousness reflects the enactment of a drive to achieve (rather than just dutifully doing what one is told), dopamine is likely to be an important influence. Achievement striving specifically is, therefore, posited to be strongly influenced by dopamine. Although at present there is little direct evidence for this hypothesis, one study found MPQ Achievement to be associated with dopamine receptor density in the midbrain and NAcc in a sample diagnosed with ADHD (Volkow et al., 2010).

### Impulsivity and sensation seeking

We now turn to traits related to dopamine that are negatively rather than positively related to Conscientiousness, and which are all related to Externalizing. Nonetheless, they are all positively related to Extraversion, and sometimes to Openness/Intellect as well. The terminology and exact definitions of these traits have been a source of confusion for decades, suffering from both the jingle fallacy (different traits called by the same name) and the jangle fallacy (the same trait called by different names). Perhaps the most confusion has been created by use of the word “impulsivity” to refer to a number of related but importantly distinct traits. Impulsivity-related constructs have been substantially clarified by the development of the UPPS model (Whiteside and Lynam, 2001; Smith et al., 2007), which identifies four distinct types of impulsivity: Urgency, lack of Perseverance, lack of Premeditation, and Sensation Seeking. Urgency, the tendency to act impulsively in ways that have negative consequences under conditions of emotional arousal, currently appears least relevant to dopamine; its major correlate in the Big Five hierarchy is low Stability (DeYoung, 2010a). Perseverance is essentially identical to Industriousness (discussed above), and thus the current theory would imply that lack of perseverance might stem in part from low global levels of dopamine (although it is also possible that a specific profile of dopaminergic responding in the value system to cues of immediate reward rather than cues of more distant reward could be responsible for lack of perseverance). The clearest evidence links lack of premeditation and sensation seeking to dopaminergic function.

Premeditation refers to “the tendency to think and reflect on the consequences of an act before engaging in that act” (Whiteside and Lynam, 2001, p 685). It is associated primarily with Conscientiousness, in the Big Five, but is more peripheral to that trait than is Industriousness/perseverance and appears to be associated almost as strongly (negatively) with Extraversion as with Conscientiousness (DeYoung, 2010a). Lack of premeditation reflects rapid action without consideration of possible negative consequences, which is perhaps the most common meaning of “impulsivity” in psychology. Its link to Extraversion suggests the degree to which Extraversion energizes behavior, presumably through dopaminergic mechanisms (Niv et al., 2007; Van Egeren, 2009). Individuals who tend not to premeditate are prone to act quickly on their exploratory impulses, rather than to engage in preliminary cognitive exploration of the possible consequences of those actions. Thus, lack of premeditation may reflect reduced activity in the dopaminergic salience system, at the same time that it reflects increased activity in the value system.

A negative association of salience system activity with lack of premeditation is plausible because of the negative association of intelligence with impulsivity (Kuntsi et al., 2004). Additionally, variation in the *DRD4* gene has been found to moderate the negative association between intelligence and the general Externalizing factor, of which impulsivity is a component (DeYoung et al., 2006). Differential functioning in value and salience systems might be particularly important in generating symptoms of attention-deficit/hyperactivity disorder (ADHD), which reflects problematic levels of impulsivity, in the form of both lack of premeditation (impulsivity and hyperactivity symptoms) and lack of perseverance (inattention symptoms). ADHD is most commonly treated by dopamine agonists, such as methylphenidate, and these appear to have their salutary effects in part by increasing dopamine in DLPFC—that is, in the salience system (Arnsten, 2006).

Sensation seeking reflects “willingness to take risks for the sake of excitement or novel experiences” (Zuckerman et al., 1993, p 759). Although it has often been considered a form of impulsivity and is associated with externalizing behavior in general (Krueger et al., 2007), a reasonable case can be made that sensation seeking is not necessarily impulsive. It may involve planning, perseverance, accurate assessment of risks, and steps taken to keep risk below a desired level (consider mountain climbing or hang gliding, for example). Indeed, although sensation seeking predicts frequency of behaviors like gambling and alcohol and drug use, it does not appear to predict problematic levels of engagement in those behaviors, whereas urgency and lack of premeditation do (Smith et al., 2007).

Although *Sensation Seeking*, *Novelty Seeking*, *Fun Seeking*, and *Excitement Seeking* all appear to reflect the same latent trait, some scales with these labels are broader than others. Zuckerman's (1979) Sensation Seeking Scale, for example, contains not only Thrill-and-Adventure-Seeking and Experience-Seeking subscales, but also Disinhibition and Boredom Susceptibility subscales, which have been found to reflect lack of perseverance more than sensation seeking in the UPPS system (Whiteside and Lynam, 2001). Cloninger's (1987) Novelty Seeking scale is similarly broad, containing subscales labeled Exploratory Excitability, Extravagance, Impulsiveness, and Disorderliness. The more pure measures of Sensation Seeking include the version from the UPPS scales (Whiteside and Lynam, 2001), Excitement Seeking from the NEO PI-R (Costa and McCrae, 1992b) and Fun Seeking from the BIS/BAS scales (Carver and White, 1994). Regardless of their breadth, all of these measures have in common that they are associated positively with Extraversion and negatively with Conscientiousness, though the balance is shifted more toward Extraversion in the purer scales (DeYoung and Gray, 2009; Quilty et al., 2013). As noted by Depue and Collins (1999), variation in impulsivity-related traits is likely to be the result not only of variation in the strength of impulses to approach rewards (related to Extraversion), but also of variation in the strength of top–down control systems that constrain those impulses (related to Conscientiousness).

Using PET to assess the binding potential of dopamine D2 autoreceptors in the SNc and VTA, Zald and colleagues have produced compelling evidence for the importance of increased dopaminergic function for lack of premeditation and sensation seeking. They have shown that both Cloninger's Novelty Seeking scale and the Barratt Impulsiveness Scale (which primarily assesses lack of premeditation; Whiteside and Lynam, 2001) predict reduced D2 binding in the midbrain, which in turn predicts greater dopaminergic release in the striatum in response to amphetamine (Zald et al., 2008; Buckholtz et al., 2010b). Because the D2 autoreceptors in the midbrain inhibit dopaminergic neurons, reduced binding potential translates to greater dopaminergic activity. These results are consistent with previous research associating dopaminergic function with sensation seeking and impulsivity (Zuckerman, 2005).

Whether the salience system, as well as the value system, is involved in sensation seeking seems likely to depend on exactly what type of sensation is being sought. If sensation seeking involves planning and forethought (e.g., mountain climbing, hang gliding), then it may be associated with increased activity in the salience system, whereas more spontaneous sensation seeking seems less likely to be related to salience. The effect of dopamine on behavior can either facilitate long-term goal pursuit or hinder it, depending on other factors that are likely to include not only the ability of DLPFC to maintain a stable focus on long-term goals but also differential influence of different parts of the dopaminergic system (value vs. salience, striatal vs. cortical, tonic vs. phasic). This observation may account for the fact that some Extraversion-related traits are positively related to Conscientiousness, whereas others are negatively related.

### Aggression

Aggression is another trait, like lack of premeditation, that might be influenced in opposite directions by the value and salience systems. Salience system deficits are suggested by the negative association of working memory and intelligence with aggression (Seguin et al., 1995; Koenen et al., 2006; DeYoung et al., 2008; DeYoung, 2011). However, more direct evidence is available for the positive association of the value system with aggression. Buckholtz et al. (2010a) found that a trait of Impulsive Antisociality (combining rebelliousness, impulsivity, aggression, and alienation) was associated with dopaminergic response to amphetamine, even after controlling for impulsivity, novelty seeking, and Extraversion (notably, this was in the same sample in which they also showed associations of dopaminergic function with novelty seeking and impulsivity). These results are reasonably congruent with animal studies linking dopamine to aggression (Seo et al., 2008), and to studies reporting high levels of dopaminergic metabolites (and low levels of serotonin metabolites) in highly aggressive populations (Soderstrom et al., 2001, 2003). Like most externalizing behaviors other than sensation seeking, aggression is probably more strongly related to serotonergic than dopaminergic function, but dopamine nonetheless seems likely to be an important secondary influence.

Aggression is an excellent indicator of the low pole of Agreeableness, and specifically of the Politeness aspect of Agreeableness that is negatively related to Assertiveness, such that they form adjacent axes of the interpersonal circumplex, as depicted in Figure 2 (DeYoung et al., 2013b). This link to Assertiveness suggests that aggression is facilitated by activity in the value coding dopaminergic system. Assertive people may be more willing to take aggressive action to pursue rewards. One important consideration in the possible association of dopamine with trait levels of aggression is the difference between reactive and proactive aggression, which have different biological substrates (Lopez-Duran et al., 2009; Corr et al., 2013). Reactive or defensive aggression is aimed at eliminating a threat, often appears with panic, and is controlled by low-level defense systems in the brain that are inhibited by serotonin (Gray and McNaughton, 2000). Proactive or offensive aggression is aimed at acquiring resources, dominance status, or revenge and seems more likely to be influenced by dopamine. (Of course, individual acts of aggression may reflect a blend of reactive and proactive that is difficult to disentangle.) A study comparing rats bred to be either high or low in threat sensitivity found that both groups were more aggressive than normal rats, but that dopaminergic antagonists applied to the NAcc reduced aggression only in the low threat-sensitivity rats whose aggression seems likely to be offensive rather than defensive (Beiderbeck et al., 2012).

### Depression and anxiety

The next traits considered are those that may be negatively related to dopaminergic function in both value and salience systems. These fall within the aspect of Neuroticism labeled Withdrawal, which is one of two traits strongly linked to Plasticity that fall outside of Extraversion and Openness/Intellect in the Big Five hierarchy (the other being Industriousness). The grouping of depression and anxiety in a single trait dimension is consistent with clinical research showing that risks for diagnosis of depression and generalized anxiety disorder overlap very strongly, forming a more general factor that has been labeled “Distress” (Wright et al., 2013). In the Big Five hierarchy, Distress is equivalent to Withdrawal. (Note that, in the PID-5, a slightly different factor is labeled Withdrawal, which represents *social* withdrawal specifically, rather than anxiety and depression; De Fruyt et al., 2013.) The connection of the Withdrawal aspect of Neuroticism with low Plasticity is consistent with lexical research, in which the Dynamism factor that appears when only two factors are extracted is related to Withdrawal (Saucier et al., 2013). An absence of depressed or anxious affect appears to be importantly related to Plasticity.

Neuroticism is considered to reflect the primary manifestation in personality of sensitivity to threat and punishment. In Gray's system, Neuroticism is the result of the joint sensitivities of the BIS and the FFFS (Gray and McNaughton, 2000; Corr et al., 2013). The FFFS produces active avoidance (panic, defensive anger, and flight) in response to threats where the only motivation is avoidance. Variation in FFFS sensitivity is not hypothesized to be related to dopamine. The BIS produces passive avoidance, inhibiting behavior and increasing vigilance and arousal when there is conflict between multiple possible goals or representations—in other words, in response to increases in psychological entropy. The prototypical activator of the BIS is an approach-avoidance conflict, in which the possibility of some reward is juxtaposed with the possibility of punishment (for example, when the desire to meet a potential mate is in conflict with the fear of rejection). The BIS operates by inhibiting approach toward the goal in question. In other words, it is antagonistic to the BAS, suggesting BIS sensitivity may be negatively associated with activity in the dopaminergic system. The BAS is inhibited by the BIS in order to produce caution that can prevent encountering the danger potentially associated with the current goal (Gray and McNaughton, 2000). In the Big Five hierarchy, BIS sensitivity seems to correspond to Withdrawal (DeYoung et al., 2007; Corr et al., 2013). Gray and McNaughton (2000) subdivide the passive avoidance states associated with the BIS into anxiety and depression, based on whether the danger in question is perceived to be avoidable or unavoidable. Passive avoidance in general is a response to dangers that must be approached in order to achieve some goal. When one is anxious, approach is slowed, caution and vigilance are increased, and arousal increases to prepare for a possible switch to flight or panic controlled by FFFS, if danger becomes too great. Anxiety is a state in which the possibility of punishment has not entirely overcome the possibility of reward, such that the goal in question is still potentially attainable. In contrast, depression is a state in which punishment is perceived to be unavoidable, which can be described cybernetically as a state in which a goal (and therefore reward) is perceived to be unattainable. Anxiety can be alleviated either by determining that no real threat is present or by acting in such a way as to eliminate the threat or at least to reduce the likelihood of punishment. Alternatively, anxiety can be alleviated by abandoning the operative goal and turning to some other goal (cf. Nash et al., 2011). If the previously operative goal is not soon replaced by another goal, this abandonment becomes equivalent to entering a state of depression. Depression is typically identified when this amotivated state is persistent across situations and generalizes to multiple goals. When depression is used to describe a clinical condition, then the abandonment of goals has been inappropriately generalized. Depression has been described as “learned helplessness” to reflect the fact that motivation has been extinguished in the face of threat and the perceived difficulty of achieving goals generally (Miller and Norman, 1979).

Degree of motivation to explore the possibilities for attaining a goal, during or after passive avoidance, may be the core contribution of individual differences in dopamine to depression. That dopaminergic function is diminished in depression is well-established (Dunlop and Nemeroff, 2007). The symptom of depression most often linked to dopamine is *anhedonia*, loss of interest or pleasure in one's usual activities, and this is the feature of depression that is most clearly negatively associated with Extraversion (e.g., De Fruyt et al., 2013). Because Extraversion is the trait that reflects variation in the energetic enjoyment and pursuit of rewards, anhedonia may be essentially equivalent to low Extraversion (or perhaps low Plasticity) in conjunction with high Neuroticism. Like Extraversion, depression is related to reward sensitivity, though of course negatively rather than positively (Pizzagalli et al., 2009; Bress et al., 2012). The loss of interest associated with anhedonia is particularly likely to be associated with reduced dopaminergic function (Treadway and Zald, 2013). Loss of interest might be best described as *amotivation*, reserving “anhedonia” to describe loss of pleasure, which seems likely to be more related to the opioid liking system than to dopamine. In the present theory, the amotivation associated with depression reflects a reduction in dopaminergically driven exploration of possibilities either for reward or for information that might allow the creation of viable new goals or strategies. Both the value and salience systems thus seem likely to be influential in depression. In relation to salience, depression is associated not only with reduced motivation in general but also with cognitive deficits that may stem from reduced dopaminergic tone in DLPFC (Murrough et al., 2011).

#### Anxiety is probably related to noradrenaline but not dopamine

The association of anxiety with dopaminergic function is more uncertain than that of depression, and any associations found between anxiety and dopamine may be due to the high correlation between anxiety and depression. Future research needs to disentangle these related traits carefully (cf. Weinberg et al., 2012). Little evidence links dopamine to trait anxiety or anxiety disorders specifically. Several candidate gene studies have reported associations of various dopaminergic genes with anxiety or the broader trait of Neuroticism, but, in addition to the fact that they typically did not control for depression, they may be false positives, given the lack of confirming evidence from genome-wide association studies (e.g., de Moor et al., 2010). Amotivation, which provides the clearest evidence for dopamine's involvement in depression, is not a central feature of anxiety. The present theory takes the position that anxiety, as a trait distinct from depression, is unlikely to be related to individual differences in dopaminergic function.

As preliminary and indirect evidence for this hypothesis, Table 1 presents analyses of associations between depression and anxiety and traits from the Big Five hierarchy depicted in Figure 1, assessed in 481 members of the ESCS. Anxiety and depression were measured using the NEO PI-R, which has no items identical to those in the questionnaires used to measure the Big Five and their aspects (BFAS) or the metatraits, which were assessed using the 40 items previously identified as specific markers of Stability or Plasticity (DeYoung, 2010c). Although at the zero order anxiety was correlated with most of the traits hypothesized to be influenced by dopamine, this was due to the variance anxiety shares with depression. After controlling for depression, anxiety was not significantly correlated with any of the traits in question (except of course Withdrawal, of which it is a facet). Depression, in contrast, remained correlated with those traits after controlling for anxiety. (The only exceptions for depression were Openness/Intellect and Openness, which are to be expected because Openness is positively related to Neuroticism, despite the fact that Intellect is negatively related; DeYoung et al., 2012). What this pattern suggests is that, although dopaminergic function may be negatively associated with Withdrawal, which represents the general tendency toward passive avoidance, only depression is likely to be associated with dopamine once one examines variance specific to anxiety or depression. If one considers anxiety without controlling for depression, however, anxiety may appear negatively associated with dopaminergic function.

**Table 1:** **Associations of NEO PI-R Anxiety and Depression (Costa and McCrae, 1992b) with the Big Five aspect scales (DeYoung et al., 2007) and Plasticity and Stability scales (DeYoung, 2010c) in the Eugene-Springfield community sample**.

	**Correlations**		**Partial correlations**	
	**Anxiety**	**Depression**	**Anxiety**	**Depression**
Plasticity	−0.23^*^	−0.35^*^	0.01	−0.27^*^
Stability	−0.53^*^	−0.68^*^	−0.13^*^	−0.52^*^
Extraversion	−0.24^*^	−0.40^*^	0.04	−0.33^*^
Enthusiasm	−0.18^*^	−0.33^*^	0.06	−0.28^*^
Assertiveness	−0.22^*^	−0.35^*^	0.02	−0.28^*^
Openness/Intellect	−0.05	−0.07	0.00	−0.05
Intellect	−0.18^*^	−0.20^*^	−0.06	−0.11^*^
Openness	0.11^*^	0.10^*^	0.06	0.04
Neuroticism	0.70^*^	0.71^*^	0.42^*^	0.46^*^
Withdrawal	0.73^*^	0.76^*^	0.46^*^	0.52^*^
Volatility	0.51^*^	0.51^*^	0.26^*^	0.27^*^
Agreeableness	0.00	−0.10^*^	0.09	−0.14^*^
Compassion	0.08	−0.05	0.15^*^	−0.13^*^
Politeness	−0.09	−0.13^*^	0.00	−0.10^*^
Conscientiousness	−0.09	−0.25^*^	0.12^*^	−0.26^*^
Industriousness	−0.25^*^	−0.42^*^	0.04	−0.34^*^
Orderliness	0.10^*^	−0.01	0.15^*^	−0.10^*^

N = 481,

* p < 0.05

Having staked out the position that trait anxiety is unrelated to dopamine, except inasmuch as it is related to trait depression, I now discuss potential evidence against this position, with the caveat that it comes from rodent research, so generalization to humans is uncertain. One study showed decreased exploration and increased postural indicators of anxiety in rats following depletion of dopamine in medial PFC (Espejo, 1997). A more recent study in mice provides evidence that the salience system specifically might be influential in trait anxiety: A manipulated genetic deactivation of the dopaminergic system in response to aversive events was found to lead to failure to learn about specific threats, which in turn led to an overgeneralized threat-sensitivity analogous to generalized anxiety (Zweifel et al., 2011). Thus, failure to learn, due to reduced salience system activity, might lead to anxiety due to increased psychological entropy (i.e., increased uncertainty).

Nonetheless, it is possible that dopaminergic activity in the salience system under aversive conditions is orthogonal to anxiety if the latter is considered independently of depression (which would be difficult to accomplish in rodents). In this case, variation in the salience system in response to threat would merely influence the likelihood that someone who responds with anxiety will engage in active or “problem-focused” coping (cf. Carver and Connor-Smith, 2010). Individuals high in anxiety with relatively high levels of dopamine should be more likely to overcome the inhibition that accompanies anxiety, in order to explore the threat in question, to explore possible solutions to the problem posed by the threat, and to rapidly begin approaching some other goal if their anxiety is great enough to produce complete passive avoidance of the goal in question. On the whole, they should have better outcomes following stress and should be less likely to transition from anxiety to depression, but they should not necessarily feel any less anxious about threat. Both noradrenaline and dopamine are released in response to stress (Schultz, 2007; Robbins and Arnsten, 2009), and the current theory proposes that proneness to anxiety under stress is related to variation in noradrenergic function, whereas proneness to active coping vs. depressive response to stress is related to variation in dopaminergic function. Under this hypothesis, higher levels of dopaminergic activity will not make people feel less anxious but will make them more likely to engage in active coping (which may lead to better outcomes and hence, indirectly, to less anxiety in the long run).

In a previous article, I proposed that the exploration associated with Plasticity “is distinct from the kind of exploration, triggered by threat that consists of vigilance and rumination designed to scan for further threat” (DeYoung, 2010c, p 27), but I now suspect that this statement needs to be qualified. Although it is likely to be the noradrenaline associated with anxiety that primarily triggers vigilance and rumination, the type of exploration associated with Plasticity may nonetheless be evoked by threat, inasmuch as the dopaminergic salience system is activated. In fact, it may be precisely those high in Plasticity who are likely to be resilient in the face of threat because increased dopaminergic activity will incline them to engage in active coping. Further, if the dedication of cognitive resources to exploring a problem (presumably driven by the dopaminergic salience system) is experienced as rumination, then salience system activity might be positively related to rumination specifically. Anxiety certainly interrupts the function of the higher cognitive systems that are facilitated by the salience coding system, but that does not necessarily mean it inhibits them (Fales et al., 2008). It may simply redirect them to consider threat, which would be consistent with the fact that the salience coding system is triggered by unpredicted aversive stimuli.

### Hypomania

While considering the role of dopamine in depression, it is important to consider hypomania, a personality trait specifically involved in bipolar or manic depression. Much as “depression” can be used to describe a personality trait as well as the more severe and typically more time-limited pathological episodes that receive a clinical diagnosis of depression, “hypomania” can be used to describe the milder and more stable personality trait that constitutes risk for episodes of mania (the prefix “hypo” indicates behavior less severe than full-blown mania). Mania is linked to heightened exploratory behavior (Perry et al., 2010), positive emotion (Gruber, 2011), and dopaminergic function (Park and Kang, 2012), and individuals described as hypomanic show behavioral signs of frequent intense activation of both value and salience systems, vividly illustrated by items from the Hypomanic Personality Scale (Eckblad and Chapman, 1986): “I have often been so excited about an involving project that I didn't care about eating or sleeping” (value); “Sometimes ideas and insights come to me so fast that I cannot express them all” (salience).

Consistent with involvement of both divisions of the dopaminergic system, trait hypomania is positively associated with both Extraversion and Openness/Intellect (Meyer, 2002; Schalet et al., 2011). Similarly, diagnosis of bipolar disorder is associated with elevated Extraversion and Openness/Intellect, a very unusual pattern among psychiatric disorders (Tackett et al., 2008). The link to general dopaminergic function is additionally consistent with the fact that mania has been linked to achievement striving (Johnson, 2005). Finally, for the salience system to be hyperactive in hypomania would be consistent with the former's apparent role in positive schizotypy, given that bipolar and schizophrenia-spectrum disorders share considerable genetic risk (Craddock and Owen, 2010). Whereas unipolar depression and depression as a personality trait are posited to be associated with a general reduction in dopaminergic function, mania and hypomania are posited to reflect a strong general increase in dopaminergic function. The neurobiological dynamics that induce alternating episodes of reduced and hyperactive dopaminergic function constitute one of the most important topics for future research on bipolar disorder and related traits.

## Summary of dopaminergic traits and conclusion

Table 2 presents the list of traits hypothesized to be influenced by dopamine, noting whether each is hypothesized to be primarily or secondarily associated with the value or salience coding dopaminergic systems. A primary association indicates that variation in the particular dopaminergic subsystem is hypothesized to be one of the largest determinants of variation in the trait. A secondary association indicates that other biological systems are hypothesized to determine more variance in the trait than does the particular dopaminergic subsystem. The sign of the association indicates whether dopaminergic activity is positively or negatively related to trait level. Activity in the value system influences traits that mainly involve behavioral exploration, whereas activity in the salience system influences traits that mainly involve cognitive exploration (taking a broad definition of “exploration” as any process that functions to transform the unknown into the known or vice versa). Traits linked to the value coding system are related to Extraversion and its subtraits; traits linked to the salience coding system are related to Openness/Intellect and its subtraits. Aggression and some forms of impulsivity (particularly lack of premeditation) are unusual in that they are posited to be positively associated with activity in the value system but negatively related to activity in the salience system.

**Table 2:** **Traits hypothesized to be related to the value coding and salience coding dopaminergic systems**.

	**Value coding**	**Salience coding**
Plasticity (Exploration)	++	++
Extraversion	++	
Assertiveness	++	
Enthusiasm	+	
Openness/Intellect		++
Intellect		++
Openness		+
Intelligence		+
Creativity	(+)	++
Apophenia (Positive schizotypy)		++
Industriousness (Perseverance)	+	+
Achievement striving	++	++
Sensation seeking	++	(+)
Impulsivity (lack of premeditation)	+	−
Aggression (low Politeness)	+	−
Depression (facet of Withdrawal)	−	−
Hypomania	++	++

++, Primary positive influence; +, Secondary positive influence; −, Secondary negative influence; parentheses indicate association conditional on different forms of the trait in question.

The present theory has several implications for research on the role of dopamine in personality. First, the difference between value and salience systems clarifies one major reason why not every measured parameter of dopaminergic function must be related to every dopaminergic trait. Some traits will be related to parameters specific to one or the other system. Second, even within each system, different parameters may be related to different traits (because of the complexity of each system and their interactions with each other). For example, a dopaminergic value-system parameter that predicts sensation seeking need not necessarily predict Extraversion. What should be the case, however, is that some parameter of the value system could be found that is related to both Extraversion and sensation seeking—because the theory presumes that any trait influenced by dopamine will be related to Extraversion or Openness/Intellect partly through dopaminergic mechanisms. Because of the many different parameters that may vary in the dopaminergic system, Extraversion and Openness/Intellect need not account for (or fully mediate) every association of some other trait with dopaminergic function, but any trait associated with dopaminergic function should be associated with Extraversion and/or Openness/Intellect or one of their subtraits.

Because Extraversion and Openness/Intellect are considered to be the primary manifestations of dopaminergic function in personality, one should always test whether an association between a dopaminergic parameter and some other personality trait is mediated by these two traits, and particularly by their Assertiveness and Intellect aspects, which are hypothesized to be most strongly related to dopamine. Further, when demonstrating an association of any phenomenon with Extraversion or Assertiveness, one should always test whether the effect might be due to variance shared with Intellect, and vice versa. For example, any positive association of working memory capacity or intelligence with Extraversion is likely to be merely an artifact, due to the association of these cognitive abilities with Intellect (DeYoung et al., 2005, 2009, 2013b).

The list of traits in Table 2 is intended to be reasonably comprehensive. Some of these traits may be fractionated further into facets, but all facet-level traits related to dopamine are likely to be facets of one of the traits in the list. If additional traits are identified that cannot be considered a facet of one of the traits in Table 2, they should nonetheless be related to Extraversion or Openness/Intellect. One might predict, for example, that sociosexual orientation (i.e., desire for many short-term vs. few long-term sexual relationships; Simpson and Gangestad, 1991a) is likely to be associated with dopaminergic function. Whether or not this trait qualifies as a facet of Extraversion, it is substantially correlated with Extraversion (Simpson and Gangestad, 1991b) and seems likely to be influenced by the dopaminergic value system.

One should not fall victim to the jangle fallacy and assume that because a scale has a different name it cannot be measuring one of the traits already on the list. For example, the MPQ, which is often used in research on dopamine, contains a Social Potency that is a good measure of Assertiveness (DeYoung et al., 2013b). Similarly, Novelty Seeking and Excitement Seeking are not listed because they are subsumed by Sensation Seeking.

Another important caveat is that variations in the dopaminergic system are not presumed to be solely responsible for variation in any of the traits listed here. Even traits like Assertiveness and Intellect that are hypothesized to be strongly influenced by dopaminergic function are undoubtedly influenced by non-dopaminergic neurobiological parameters as well. Further, because multiple biological systems will influence most, if not all, traits, the mere fact that a trait is associated with Extraversion or Openness/Intellect does not guarantee that it is influenced by dopamine. Some other biological system or process may be responsible for the trait associations in question.

In recent years, the most prominent theory of the role of dopamine in personality has linked it to Extraversion, reward sensitivity, and approach behavior (Depue and Collins, 1999). Recognition of the distinction between the value and salience coding systems provides a coherent framework for understanding how traits related to cognitive function, like Openness/Intellect and positive schizotypy, might also be related to dopamine. The most important premise for the development of a unified theory of dopaminergic function is that information has innate reward value, just as do food, warmth, sex, affiliation, and status. This premise allows the identification of exploration—cognition and behavior motivated by the incentive reward value of uncertainty—as the basic function of all dopaminergic activity. In turn, this unity of function may help to explain why Extraversion (sensitivity to specific rewards) and Openness/Intellect (sensitivity to the reward value of information) are sufficiently correlated to allow characterization of a higher-order Plasticity factor. Global variations in dopaminergic tone across the value and salience systems are posited to produce variation in the general exploratory tendency reflected in individual differences in Plasticity.

This theory about the nature of dopaminergic function and its role in personality is an extension of the entropy model of uncertainty (EMU; Hirsh et al., 2012), which characterizes anxiety as a response to uncertainty, defined as psychological entropy. What the initial presentation of EMU left out was an account of the fact that uncertainty is not only innately threatening, but also innately promising (Peterson, 1999). Uncertainty or the unknown is the only class of stimuli to have this inherently ambivalent motivational significance (Gray and McNaughton, 2000). A fully elaborated EMU can account not only for the response to entropy as a threat but also for the response to entropy as a potential source of reward. Traits related to dopamine reflect variation in the ways that individuals respond to the incentive reward value of uncertainty.

### Conflict of interest statement

The author declares that the research was conducted in the absence of any commercial or financial relationships that could be construed as a potential conflict of interest.

## Footnotes

^1^This claim may raise a red flag for those familiar with the conceptual distinction between *exploration* and *exploitation* (e.g., Frank et al., 2009). In the section *Exploration: Motivation and Emotion Associated with Dopamine*, I argue that exploratory processes, facilitated by dopamine, occur during behavior typically described as “exploitation.”

^2^In the decision-making literature, uncertainty is sometimes distinguished from ambiguity, where uncertainty describes any outcome with a known probability less than 100% and ambiguity describes events in which the exact probability of a given outcome is unknown. In the present work, I do not distinguish uncertainty from ambiguity; situations in which probabilities are unknown are more uncertain than situations in which probabilities are known. Further, from the perspective of psychological entropy, a situation can contain observable uncertainty or ambiguity that is deemed neutral or irrelevant and is, therefore, *not* uncertain from the perspective of the cybernetic system because it is predicted. For example, one might observe that a particular event of no consequence takes place with uncertain frequency. That event would often be treated as minimally (if at all) unpredicted. (Consider, as an example, the variability in the noises made by one's refrigerator).

^3^The neuromodulators dopamine, noradrenaline, and acetylcholine all appear to govern elements of adaptation to increases in psychological entropy (Yu and Dayan, 2005; Hirsh et al., 2012), whereas serotonin appears to govern the stabilization of goal-directed behavior that allows avoidance of increased entropy; the latter is accomplished by serotonin's suppression of disruptive impulses and facilitation of goal-congruent behavior (Gray and McNaughton, 2000, Appendix 10; Carver et al., 2008; DeYoung, 2010a,b; Spoont, 1992).

^4^A six-factor solution may be somewhat more replicable across languages (Ashton et al., 2004), but this system is not very different from the Big Five because the major change is simply to split Agreeableness into two factors (DeYoung et al., 2007; McCrae et al., 2008; De Raad et al., 2010). At any rate, the primary traits of interest for the present theory, Extraversion and Openness/Intellect, remain essentially the same in the six-factor solution.

^5^Note that the reward learning associated with the dopaminergic value system, which the present theory associates primarily with Extraversion, can be considered a basic form of “cognitive exploration.” However, the potentiation of exploration that would more typically be considered “cognitive,” involving the search for correlational or causal patterns in perception and memory, is posited to be the function of the salience system and hence associated with Openness/Intellect.

^6^Based on this description of the psychological meaning of Stability, one might expect it to be influenced by dopamine, given dopamine's role in the maintenance of the stability of goal representations in DLPFC. Dopamine in DLPFC is certainly important for the neural stability of representations in working memory (Robbins and Arnsten, 2009). However, no direct or indirect evidence of the sort cited for other traits in the present theory exists to suggest that dopamine influences the personality trait Stability. Traits from the Openness/Intellect domain are the only traits in the Big Five hierarchy that are consistently related to working memory performance (DeYoung et al., 2005, 2009). It may be that representations in working memory (even when they are well-stabilized by dopamine) are present for too short a time to be relevant to the kind of motivational stability reflected by the broad Stability trait. Only information currently in the field of conscious attention is maintained and manipulated by working memory. Additionally, the distractions suppressed in Stability are impulses related to reward or punishment and thus not identical to the cognitive distractions that must be suppressed for good working memory function.

^7^The path from Plasticity to Externalizing reported by DeYoung et al. (2008) was actually slightly greater than the path from Stability. However, this is likely to be a quirk of this sample and not to generalize, because externalizing behavior has typically been found to be associated considerably more strongly with Neuroticism, low Agreeableness, and low Conscientiousness than with either Extraversion or Openness/Intellect.
